# On-farm investments into dairy cow health: evidence from 15 case study countries

**DOI:** 10.3389/fvets.2023.1288199

**Published:** 2023-10-31

**Authors:** Peggy Schrobback, Carlos Gonzalez Fischer, Dianne Mayberry, Mario Herrero

**Affiliations:** ^1^Global Burden of Animal Diseases Program, Institute of Infection, Veterinary and Ecological Sciences, University of Liverpool, Liverpool, United Kingdom; ^2^CSIRO, Agriculture and Food, St Lucia, QLD, Australia; ^3^Department of Global Development, College of Agriculture and Life Sciences and Cornell Atkinson Centre for Sustainability, Cornell University, Ithaca, NY, United States

**Keywords:** dairy, costs, cow, disease, health, investment, livestock, production

## Abstract

Managing investments in dairy cow health at a national and global scale, requires an improved understanding of current on-farm expenses for cow health (e.g., expenditure for medicine and veterinary consultations). The aim of this study was to assess on-farm health investments for typical dairy farms in 15 case study countries, including Argentina, Australia, Bangladesh, Brazil, Canada, India, China, Colombia, Indonesia, Kenya, New Zealand, Uganda, UK, Uruguay, and USA. The study was conducted using a descriptive analysis of a secondary data set that was obtained from the International Farm Comparison Network (IFCN). The results suggest that health expenditures take up a relatively small proportion (<10%) of the annual total production costs per cow across all countries in the sample. The means of production costs (e.g., feed, machinery) can take up to 90% of the total production costs for highly intensive systems, while these costs can be as low as 9% for extensive systems. This study highlights the importance of understanding on-farm animal health investments as a contribution to improved national and global decision making about animal health in the dairy sector.

## 1. Introduction

The global dairy sector is an important source of protein and other nutrients that contribute to ensuring food security and nutrition, and also provides income generation opportunities for rural communities worldwide (1). Yet, the global dairy sector is experiencing a range of pressures (e.g., climate change impacting feed availability, habitat shifts and heat stress; changing consumer expectations and shifts); with the prevention, treatment, and management of diseases being one of the key challenges (2, 3).

The health of dairy cattle can impact their productivity, production profitability, zoonotic risks, international trade (e.g., biosecurity risk associated with transboundary infectious diseases), and animal welfare (4). To improve the sustainability of dairy cattle production, national and on-farm investments in dairy cattle health (e.g., biosecurity regulations and enforcement, vaccine and medicine application, herd health monitoring) are vital to prevent diseases and to manage them effectively if they occur (5).

The literature offers a range of studies which focus on the on-farm costs or expenditures for managing specific diseases in dairy cattle (e.g., lameness, mastitis, metritis, retained placenta, left-displaced abomasum, ketosis, and hypocalcemia) in selected countries [e.g., (6–10)]. There are also studies that assess the economic impact of specific dairy cattle diseases, for example, Johne’s disease (11, 12), mastitis (13), or food and mouth disease (14–16). However, there is not - to the best of our knowledge - a proper understanding about farm-scale health expenditures for dairy cattle. This need has also been identified by Perry et al. (17).

The aim of this study was to address this gap by assessing on-farm health costs for dairy cows in 15 case study countries representing a diversity of dairy production systems, including a comparison to other production costs (e.g., feed, labor), milk yields and animal losses, and its variations among different countries.

Information generated in this study is to be considered as a proof of concept, emphasizing the value of systematically collected production and animal health data at farm scale. The findings may be useful for intergovernmental organizations, national governments, dairy industry associations, and veterinarians to collaboratively address the data gaps around global farmed animal health. Insights into global on-farm animal health investments are also of interest for the Global Burden of Animal Disease (GBADs) program,^1^ as a component of the animal health loss envelope (18) which provides a baseline for assessments of the costs and benefits of investments in improved animal health to global society.

## 2. Materials and methods

### 2.1. Data material

To gain an improved understanding of current global investments into on-farm dairy cow health, a secondary data set from the International Farm Comparison Network (IFCN) was acquired. A case study approach, including 15 countries (using 2021 as the reference year), was selected to demonstrate the value of information about on-farm animal health and production data to understand global differences in animal health and disease management.

**Figure 1:**
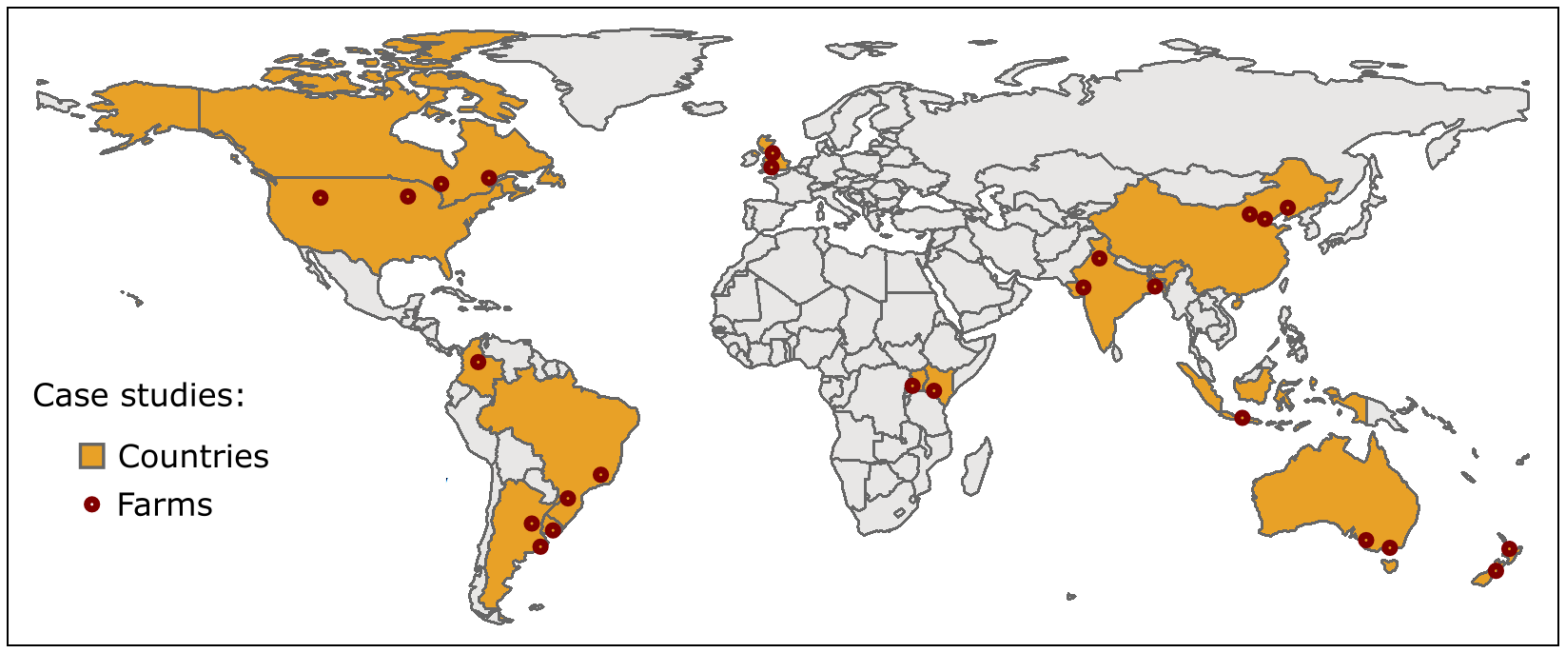
Map of case study countries and approximate data collection areas. Notes: ‘Farms’ indicate the locations within a country where the secondary data was collected.

The 15 countries were selected on the basis of: (a) availability within the IFCN database for 2021, (b) income level according to the The World Bank (19) classification (i.e., high-income, upper middle-income, lower middle-income, low-income), (b) share of global milk production, and (c) geographic region, with the aim of including a diverse range of production systems within our analysis. This resulted in the selection of the following countries: Argentina, Australia, Bangladesh, Brazil, Canada, China, Colombia, India, Indonesia, Kenya, New Zealand, Uganda, United Kingdom, United States of America (United States), and Uruguay (see Figure 1). It should be noted that Uganda was the only low-income country available in the IFCN database for 2021, which is due to the difficulties in establishing research partnerships, including collaborative data collection, in these countries (IFCN, personal communication in March 2023).

**Figure 2:**
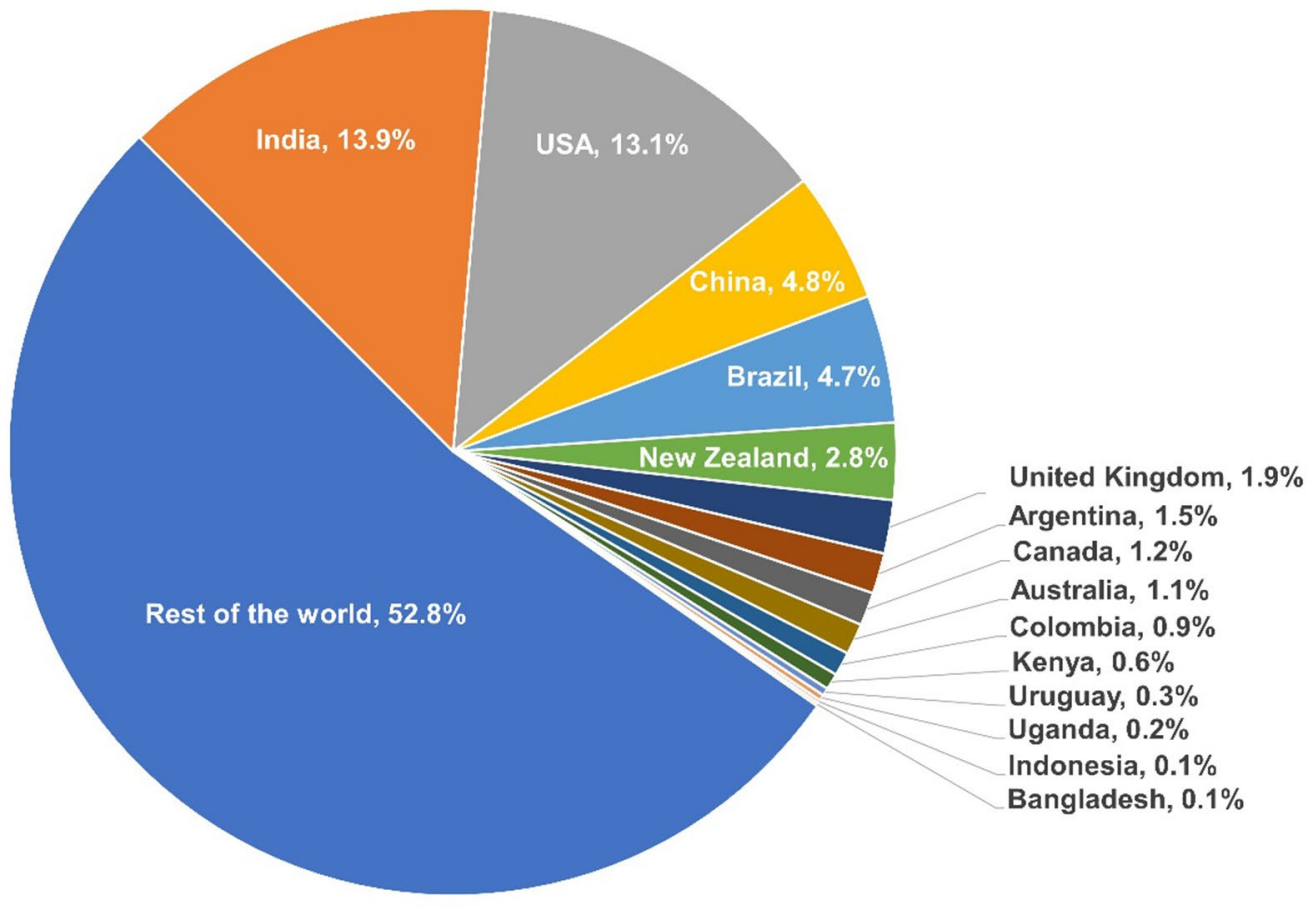
Case study country’s contribution to the total global milk production output in 2021. Source: FAO (20).

The 15 case studies countries together produced nearly half (47.2%) of the total global milk production output in 2021 as illustrated in Figure 2 (20).

For each of the 15 countries, data for two different farm types were provided in the IFCN dataset (21). For larger countries such as Brazil, China, India, and the United States, data for four farm types were available. The total number of farm types included in the analysis was 38. Importantly, these farm types were characterized by IFCN [e.g., (21)] in the context of each individual country, e.g., typical dairy farming systems observed in each country. This implies that the farm types may not be directly comparable between countries.

For each farm type, the data set included standardized annual information about dairy production systems for 2021. A wide range of production system variables that describe the farm types were available, including number of milking cows, predominant breed, average milk yield per milking cow (e.g., kilogram per cow/year), cow losses (e.g., proportion of cows died), size of farms, stocking rates, production system type, and production costs. Data about the average size of dairy land and the total land size of dairy farms was also provided, yet detailed information about the composition of these land types was not included. No information was available about the whole dairy herd size and the age structure of the herd kept on the farms, including heifers, claves, and breeding bulls.

A range of cost types were provided in the annual production data set for 2021 which we categorized into:

- means of production costs: cost of feed (feed, forage, fertilizer, seed, pesticides), machinery (maintenance, depreciation, contractor), energy and water (fuel, energy, lubricants, water), buildings (maintenance, depreciation), animal purchases, insurance taxes, other dairy enterprise inputs (e.g., milk supplies, herd testing, fees for pedigree records, bedding, fees for disease prevention board, hauling, promotion, milk quota-not used), other whole farm enterprise inputs (e.g., accounting and book keeping fees, phone and utilities costs), insemination, and value added tax balance,
- health costs (one aggregate for all types of veterinary and medicine expenses),
- land costs (one aggregate for all types of land costs, e.g., land tax),
- labor costs (one aggregate for all types of labor expenses, e.g., hired, family), and.
- capital costs (one aggregate for all types of equity and liabilities).

The cost data was provided as unit of USD/100 kg milk (solid corrected milk (SCM)). This unit cost value per farm type was multiplied with the average milk yield per milking cow which was provided as unit of kg SCM/milking cow. Information about national programs that provide free or subsidized animal health care services, e.g., medicine, vaccines, health consultations, was not given in the dataset. Annual farm gate milk price (i.e., the price farmers got paid) data was also available for each farm type represented in USD / 100 kg SCM. More detailed information about the data collection method of the original data set is described by Hemme et al. (22) and Hemme (22).

The research team considered a further disaggregation of the health cost aggregate provided in the secondary data set [see category (b) above] using expert interviews (e.g., veterinarians, dairy industry representatives, government extension officers) in each of the 15 countries. This included disaggregation of health costs into different medicine expenses, health professional consultation cost, other health costs such as surgeries, disease prevention costs and treatment costs per cow. However, when testing this method of data collection with participants in several countries (23 in total), a range of issues were identified, including difficulty in identifying knowledgeable experts who were willing to participate in interviews, and a large variance in responses for individual countries. These issues resulted in the decision to discontinue the expert interviews and subsequently the attempt to further disaggregate the health cost aggregate that was available in the IFCN data set. The Supplementary material provides information about the interview questionnaire and key learnings from the interviews, which may be of interest for the reader.

### 2.2. Methods

All costs in the data set were reported in USD, which reflect USD 2021 average exchange rate adjusted cost values that were originally collected in local currency units (LCU) by IFCN. However, for a meaningful comparison of on-farm health costs across countries, these values needed to be adjusted by the purchasing power parity (PPP) (23). The purchasing power varies greatly in different countries (e.g., the amount of feed that farmers can purchase with 100 USD is different in Uganda compared to the United States) which can lead to misinterpretation of cost and price differentials, especially when comparing absolute production costs and milk prices. To adjust the production cost and milk price data for PPP, they were first converted back into their LCU (e.g., USD back to Ugandan Schilling) using average annual market exchange rates of LCUs to the USD, and then normalized by the PPP conversion rates provided by The World Bank (24) (see in the Supplementary material Table S1).

The data set only offered observations for one production year, i.e., 2021, and 2–4 observations for farm types per country which limited the data assessment to a descriptive analysis. This included an assessment of the absolute and proportional on-farm dairy production costs based on the cost components: means of production (e.g., feed, machinery, fuel, labor, veterinary and medicine, insemination, buildings, other costs), labor, land, capital, and health. For example, the proportion of health costs*, C_H_*, compared to the total production costs, *C_T_*, can be expressed as:

(1) $$\frac{C_{H}}{C_{T}}$$

Furthermore, total production costs and health costs per cow *per annum* were analyzed based on the average milk yield per cow, *Y*, represented, respectively, by:

(2) $$\frac{C_{T}}{Y}\mathrm{and}\;\frac{C_{H}}{Y}\text{.}$$

The analysis considered the identification of trends in on-farm expenditures by the income category of countries using the The World Bank (19). The country categories were: Low-income country included Uganda, lower middle-income countries included Bangladesh, India, Indonesia, Kenya, upper middle-income countries included Argentina, Brazil, China, Colombia, high income countries included Australia, Canada, New Zealand, United Kingdom, United States, and Uruguay.

An analysis by agri-ecological zone and production classification system was also considered, but ultimately not carried out. The small number of countries included in this analysis and the lack of precise location information where the dairy farm data was collected would have limited the relevance of such analysis.

## 3. Results

### 3.1. Description of dairy farms

An overview of the key variables that describe the data for the production systems in the 15 case study countries is presented in Table 1. Different farm types for each country are identified using the codes A-D, and we reiterate that these farm types may not be comparable between countries. The number of milking cows across farm types varies significantly within the data set, e.g., 2–2,600. Holstein Friesian (HF) was the most common cow breed observed, yet other dairy breeds (e.g., Jersey), dual purpose breeds (e.g., Ankole) and crossbreds were also present.

**Table 1:** Descriptive statistics of case study countries’ dairy production systems.

Country	Country category	Farm type	Average number of milking cows per farm	Predominant breed	Proportion of farm area used for dairy production (%)	Stocking rate (cows/ha)	Average milk yield (kg SCM/cow/year)	Production system	Selected references describing county’s dairy sector
Argentina	UMI	A	180	HF	100	1.2	5,020	GF	Lazzarini et al. (25)
		B	400	HF	100	1.5	6,308	GF	
Australia	HI	A	307	HF	75	1.9	6,465	GF	Sheng et al. (26), Dairy Australia (27)
		B	420	HF	75	2.5	7.338	GF	
Bangladesh	LMI	A	2	Local	16	23.0	927	SSF	Datta et al. (28), Uddin et al. (29), Hossain et al. (30)
		B	14	Local x Shahiwal, HF	20	25.1	1,262	SSF	
Brazil	UMI	A	34	HF	100	1.5	6,995	FF	Balco et al. (31), Daros et al. (32)
		B	64	HF	72	2.9	8,001	FF	
		C	180	HF	100	1.1	4,563	GF	
		D	320	HF	100	1.2	4,969	GF	
Canada	HI	A	66	HF	93	1.6	9,117	SBF	Mc Geough et al. (33), van Kooten (34), Charlebois et al. (35)
		B	140	HF	35	1.1	9,705	FSBF	
China	UMI	A	320	HF	0	0	7,252	FSBF	Li et al. (36), Huang et al. (37)
		B	1,828	HF	0	0	11,662	FF	
		C	289	HF	0	0.0	9,023	FF	
		D	2,250	HF, Jersey	0	0.0	10,381	FF	
Colombia	UMI	A	6	HF	100	2.8	4,607	GF	Carulla and Ortega (38)
		B	108	HF	93	2.7	6,129	GF	
India	LMI	A	2	Murrah buffalo crossbred	50	2.2	4,140	SSF	Kumar et al. (39), Landes et al. (40)
		B	8	HF crossbred, Murrah buffalo	53	4.9	2,595	SSF	
		C	70	HF	70	21.0	4,385	FSBF	
		D	300	HF crossbred, Jersey	92	4.9	5,051	FSBF	
Indonesia	LMI	A	3	HF	81	1.8	3,093	SSF	Susanty et al. (41), Umberger (42), Apdini et al. (43)
		B	10	HF	99	5.4	4,060	SSF	
Kenya	LMI	A	2	HF, HF crossbreed	70	2.6	2,591	SSF	Onono et al. (44), Kibiego et al. (45), Odero-Waitituh (46)
		B	10	HF, HF crossbreed	65	2.4	2,868	SSF	
New Zealand	HI	A	380	HF x Jersey	84	2.7	5,466	GF	Dairy NZ (47), Foote et al. (48)
		B	1,171	HF x Jersey	79	3.4	6,323	GF	
Uganda	LI	A	3	HF x Ankole cattle	63	2.8	3,099	SSF	Kirunda et al. (49), Waiswa et al. (50), Waiswa and Günlü (51)
		B	13	Ankole cattle	69	3.5	679	SSF	
UK	HI	A	160	HF	94	1.7	8,403	FSBF	Arnott et al. (52), Wilkinson et al. (53)
		B	259	HF	79	1.6	7,949	FSBF	
USA	HI	A	80	HF	96	1.2	10,445	SBF	Khanal et al. (54), von Keyserlingk et al. (55)
		B	500	HF	98	1.8	11,081	FSBF	
		C	1,200	Swedish Red/HF	94	3.2	11,020	FSBF	
		D	2,600	HF	100	17.8	12,119	FSBF	
Uruguay	HI	A	129	HF	71	1.1	5,369	GF	Fariña and Chilibroste (56), Méndez et al. (57), Stirling et al. (58)
		B	367	HF	66	1.1	5,699	GF	

LI for low income, LMI of lower middle income, UMI for upper middle income, HI for high income, SCM for solid corrected milk, HF for Holstein Friesian, GF for grazing farm, SSF for small scale farm, FF for feedlot farm, SBF for stanchion barn farm, FSBF for free stall barn farm. Stocking rate refers to livestock heads per hectare of dairy land. The proportion of farm area used for dairy production was derived by the ratio of reported ‘dairy land’ size and ‘total land’ size. Details on the composition of different land types were unavailable. Source: IFCN (59).

Most farm types in the data set have a very high proportion (over 90%) of land used for dairy production proportional to the total size of farms. Lower proportions for the ratio of dairy land and total land may indicate that these are mixed system farms (e.g., dairy and cropping) which can still be grazing/pasture-based systems. There are also farm types in the sample with ‘no’ dairy land (e.g., China-A-D) which suggests a zero grazing/pasture farming system such as a feedlot but not necessarily landless dairy systems.

### 3.2. Decomposition of total production costs

**Figure 3:**
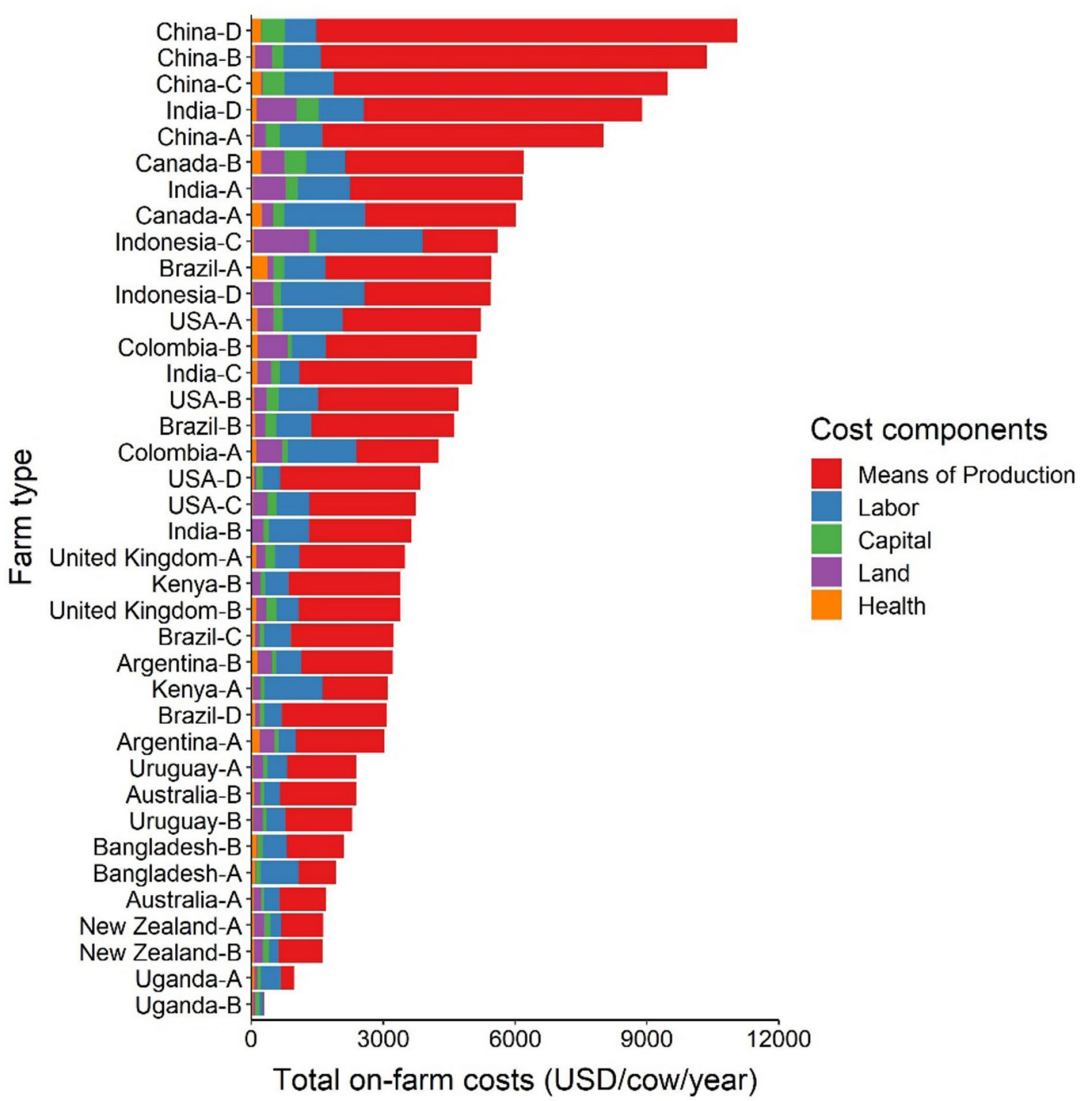
Total production costs per cow *per annum* by country, farm type and cost components. A-D for farm types available in the data set, see Table 1. Values are presented in International Dollars (i.e., USD value adjusted by PPP for each country). Source: IFCN (59).

Figure 3 presents the breakdown of the total production costs per cow *per annum* by country and farm type adjusted by PPP for cross-country comparability. Cost components include means of production (i.e., feed and forage, energy and water, machinery, building, insurance taxes, other inputs to dairy enterprise, other inputs, and VAT balance), labor, capital, land, and health (i.e., medicine and veterinary consultations) expenses.

The results indicate that the means of production costs take up the major share of the total production costs across all case study countries (median: 66.2%, ranging from 8.9–86.6%) followed by labor costs (median: 17.6%, ranging from 6.5–47.5%), capital costs (median: 4.0%, ranging from 1.9–29.7%) and land costs (median: 6.5%, ranging from 0.1–22.6%; Figure 3). The share of health costs is relatively small across all countries (median: 2.5%, ranging from 0.2–12.5%) compared to other cost components but are equal or higher than capital and land costs in selected cases (e.g., Canada-A, Brazil-A).

Three of the four Chinese dairy farm types (i.e., B, C, D) were the most cost intensive dairy systems within the data set. These farm types had a high number of cows and no dairy land (Table 1), implying that this is an intensive, zero grazing system. All feed and forage for these farm types is purchased outside the farm, which explains the high costs of means of production (80–85% of the total annual production costs).

**Figure 4:**
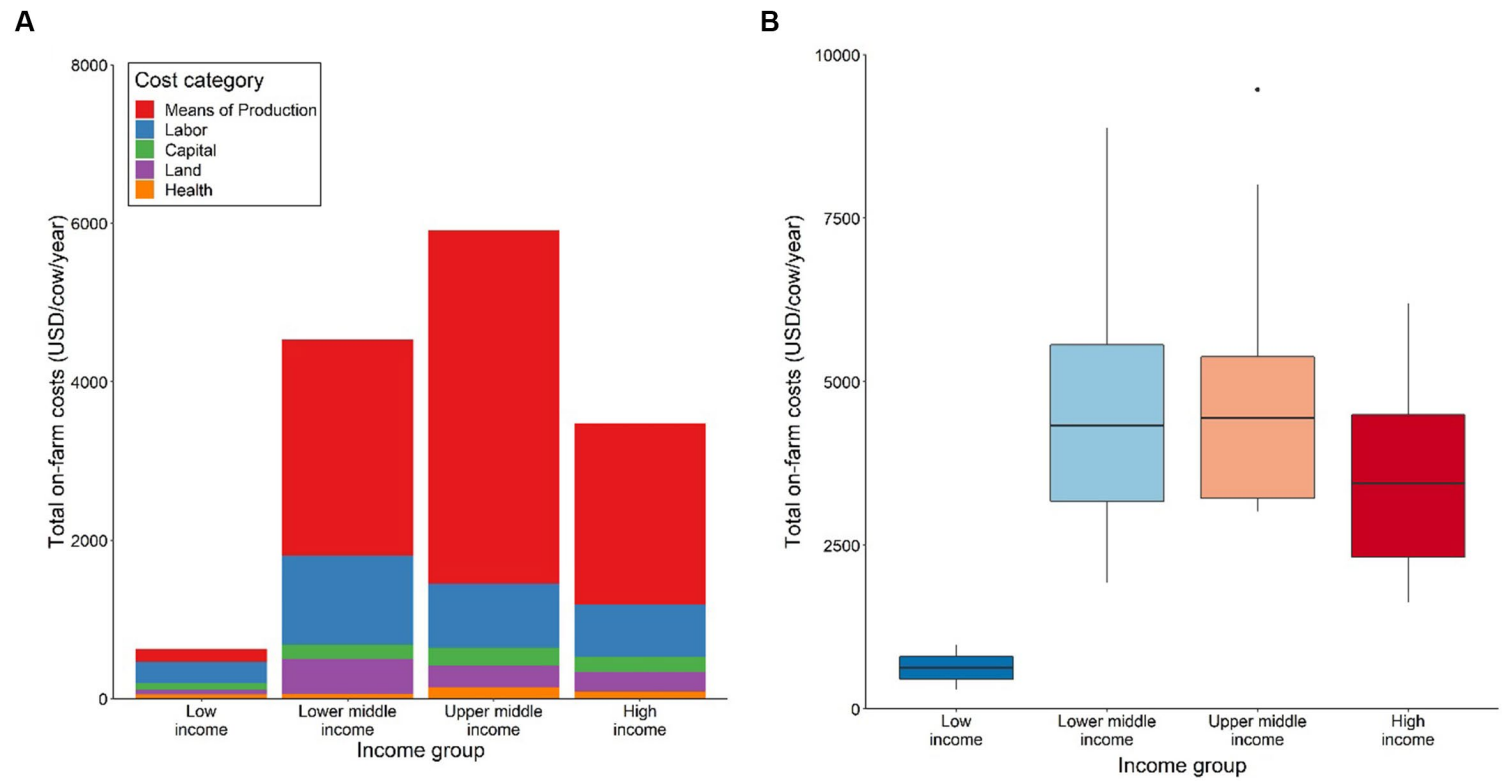
Structure of total production costs by country income group; **(A)** Average contribution of means of production, labor, capital, land, and health to total production cost, **(B)** Range of total production costs. Low-income group includes *n* = 1, lower middle-income group includes *n* = 4, upper middle-income group includes *n* = 4, high-income group includes *n* = 6. Values are presented in International Dollars (i.e., USD value adjusted by PPP for each country). Source: IFCN (59).

The results suggest that the average total on-farm costs per cow increase with the wealth of a country up to the upper middle-income category and then decreases slightly for high income countries (Figure 4). Notable is the sharp increase of average total production costs per cow from low-income to lower middle-income country category. However, as there is only one country in the data set categorized as low-income, Uganda, these results may not be representative for other low-income countries. Hence, this outcome needs to be interpreted cautiously.

The increase in average total production costs per cow with increasing wealth of a country appears to be driven by dynamics in the means of production costs across all country income categories (Figure 4). For example, upper middle-income countries appear to spend the highest average costs on means of production inputs, which then decreases for high-income countries. Average on-farm costs per cow for labor, capital, land, and health costs appear to vary only slightly across lower middle-, upper middle- and high-income countries (Figure 4A). The results also suggest that there is some variation in the range of total production costs for all income groups, except the low-income category, as shown in Figure 4B.

### 3.3. Health costs

**Figure 5:**
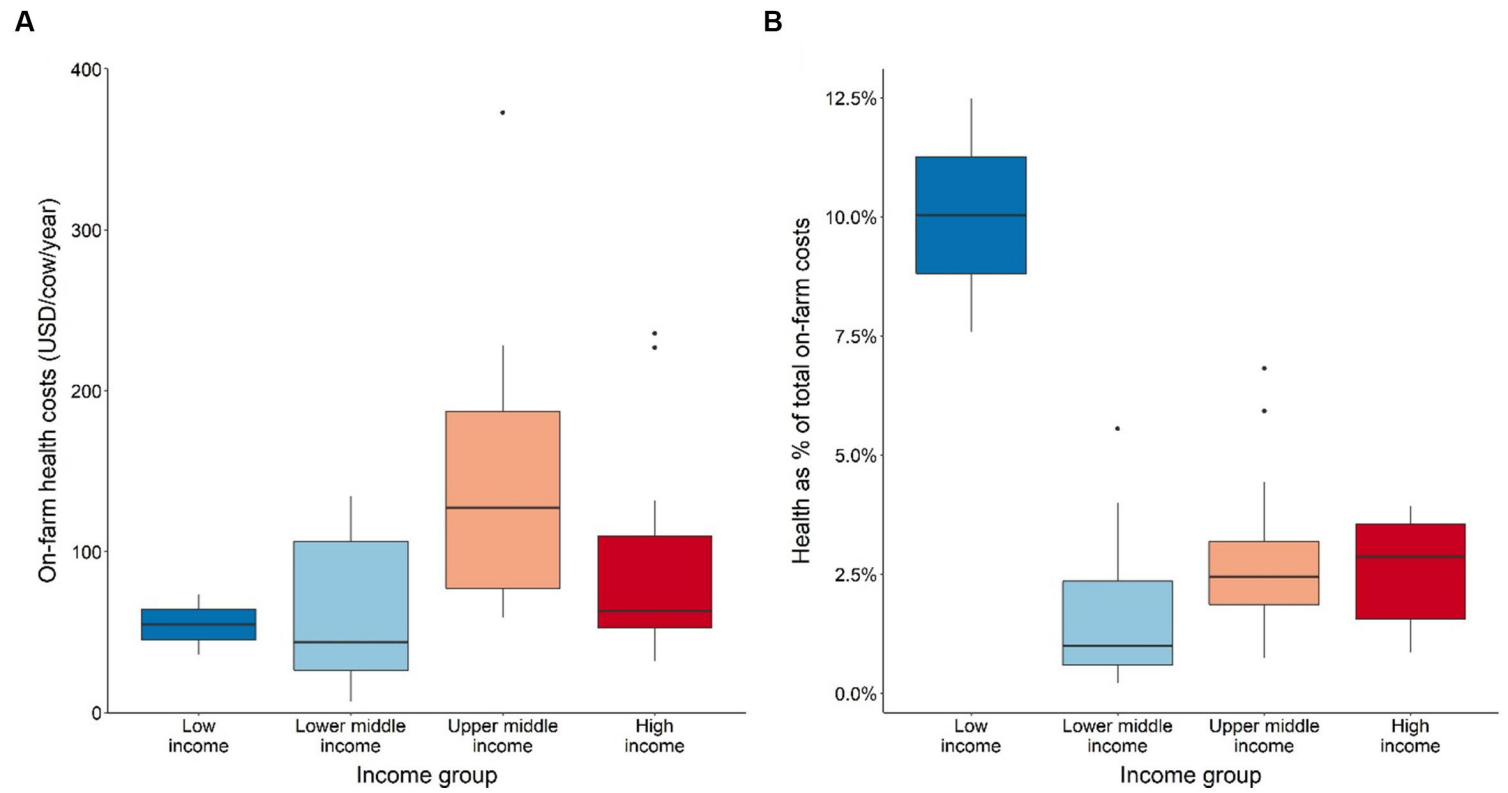
**(A)** Absolute value of on-farm health costs per cow and country income-group, and **(B)** On-farm health costs per cow as a proportion of total costs by country income group. Values are presented in International Dollars (i.e., USD value adjusted by PPP for each country). Source: IFCN (59).

The average on-farm health costs per cow range between a median of 3–250 USD *per annum* across all country income groups (Figure 5A) important data in the estimation of the animal health loss envelope in the GBADs program (18). However, the dispersion around the median values is relatively large, specifically for upper middle-income countries as indicated by the boxplots. Low-income countries spend about 10% (median) of their total production costs for animal health related expenses (Figure 5B). This proportion appears to decrease to 1.7–2.9% (median) for other country income groups.

### 3.4. Total production costs, health costs vs. milk yield

**Figure 6:**
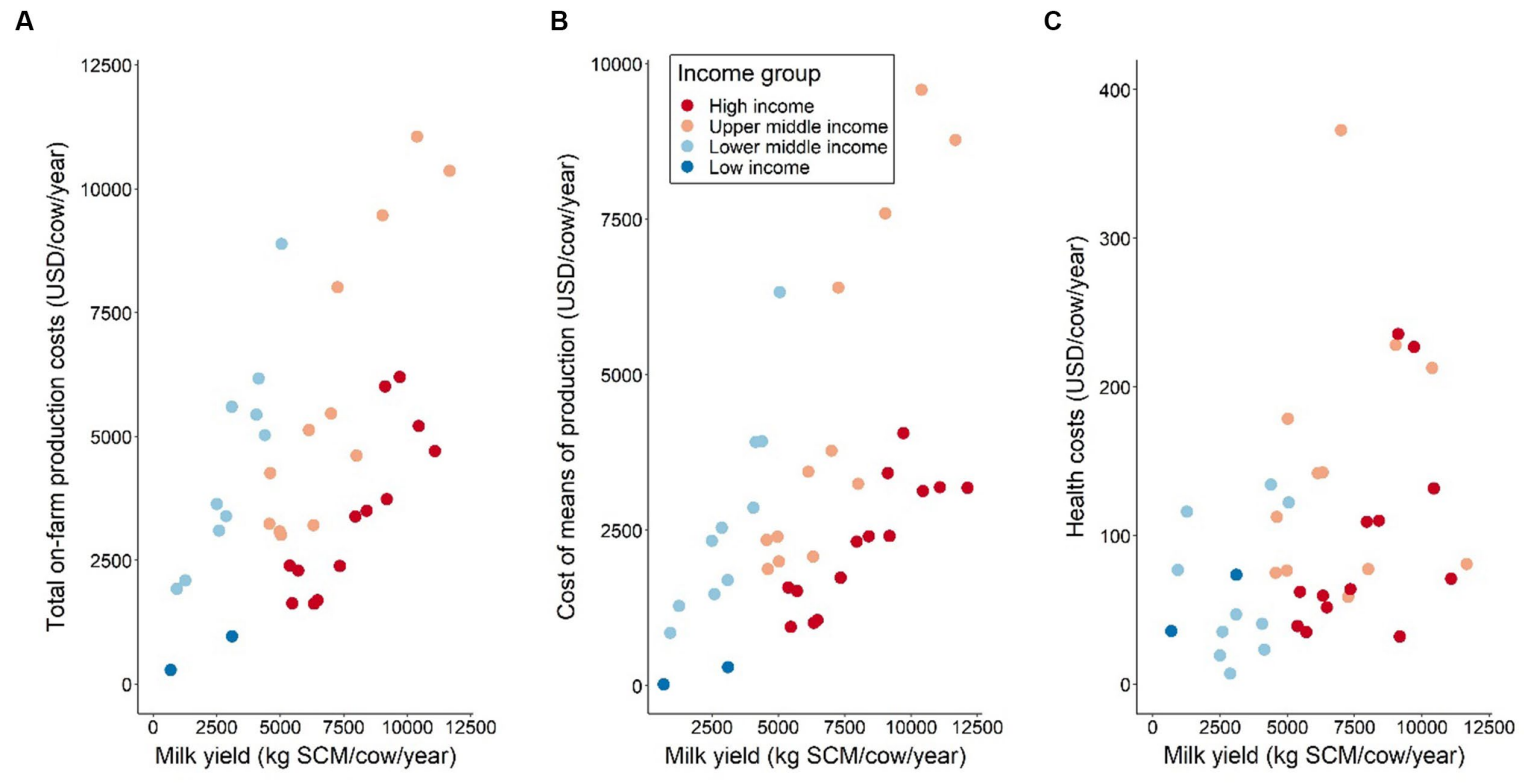
**(A)** Total on-farm production costs by milk yield and country income group, **(B)** Costs of means of production by milk yield and country income group, and **(C)** Health costs by milk yield and country income group. Values are presented in International Dollars (i.e., USD value adjusted by PPP for each country). Source: IFCN (59).

Milk yield tends to increase with rising total costs of production per cow (Figure 6A). This result may be due to higher yielding animals that are larger, require more feed and forage and are fed higher quality diets which may be more expensive. Yet, these results also show that it is more expensive for lower income countries to achieve a higher yield compared to higher income countries. This could be due to an absence of competition in input provision and subsequent higher input prices. This association is also reflected by the cost of means of production in relation to milk yield (Figure 6B), which is not surprising considering that the means of production costs contribute a major share of total production cost per cow in all farm types. Furthermore, a similar but less clear relationship can also be observed for health costs per cow and milk yield (Figure 6C).

**Figure 7:**
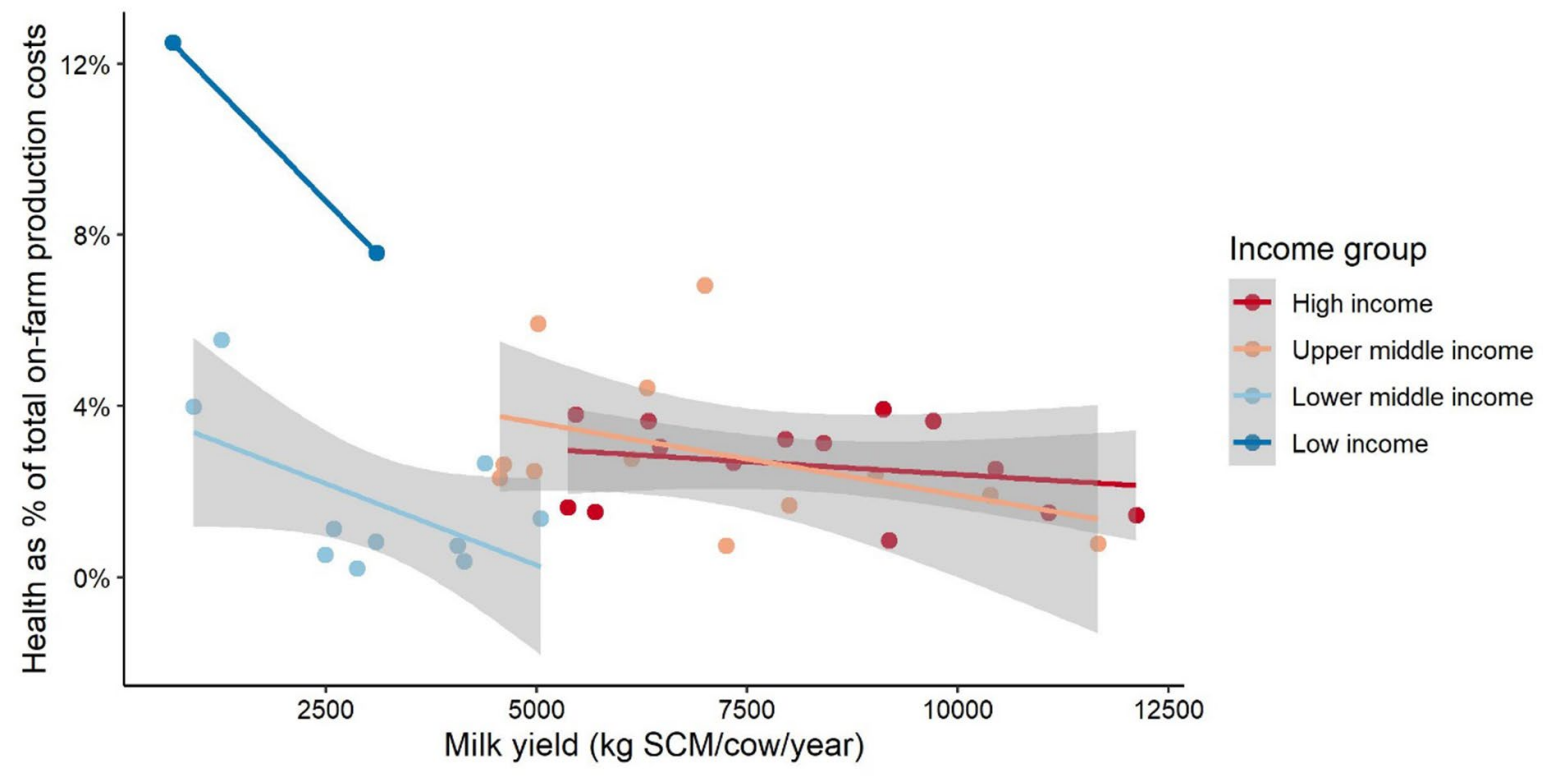
Trends in health cost–milk yield relationship. The gray area represents the confidence interval around the smoothed geometric mean. The smaller the gray area around the mean trend line, the more similar the observations within a country income group and vice versa. Cost values are presented in International Dollars (PPP adjusted values). Source: IFCN (59).

Low-income countries appear to spend a higher proportion of total production costs on animal health (Figure 7). This result is influenced by the relatively low means of production costs as a proportion of the total production costs for low-income countries (see Figure 3). This result could also be explained by the breeds used in these extensive dairy systems (e.g., local dual purpose and crossbred animals). Interestingly, there appears to be a negative relationship between the proportion of health costs investment spent of the total production costs in the milk yield for all country income categories. This result suggests that the yield of cows may be mostly associated with other input factor costs rather than health investments.

### 3.5. Total production costs, health costs vs. milk price

**Figure 8:**
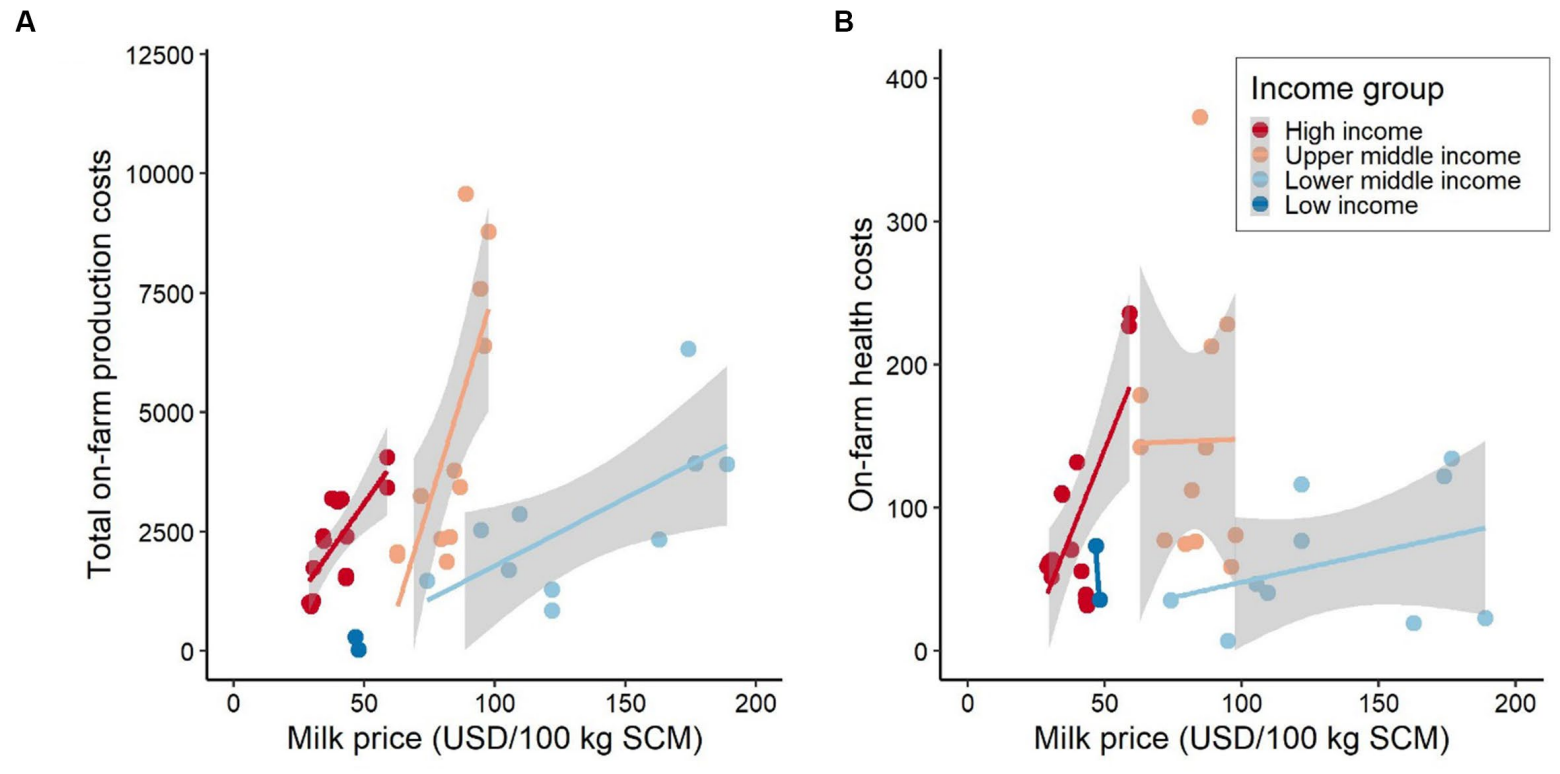
**(A)** Total on-farm production costs vs. milk price, and **(B)** health costs vs. milk price. Costs values are presented in International Dollars (PPP adjusted values). Source: IFCN (59).

The total on-farm production costs and health costs appear to rise with increasing milk price (Figures 8A,B). This relationship is evident for all country income categories, except for low-income countries, which could be due to a small sample bias, i.e., one country in this category. Yet, the causation of the positive cost–price relationships is unclear, i.e., higher production costs leading to higher milk prices, or higher milk prices leading to higher production costs. Furthermore, the results for the production cost-milk price relationship should also be interpreted with caution as the milk price in different countries can be distorted by market interventions such as subsidies. There can also be a lack of farmer’s price bargaining power which affects the milk price across and within different countries, and other financial sources may be available to farmers to cross-finance dairy production input costs (e.g., in crop-dairy production systems). Yet, such information was not available in the data.

### 3.6. Health costs vs. number of cows

**Figure 9:**
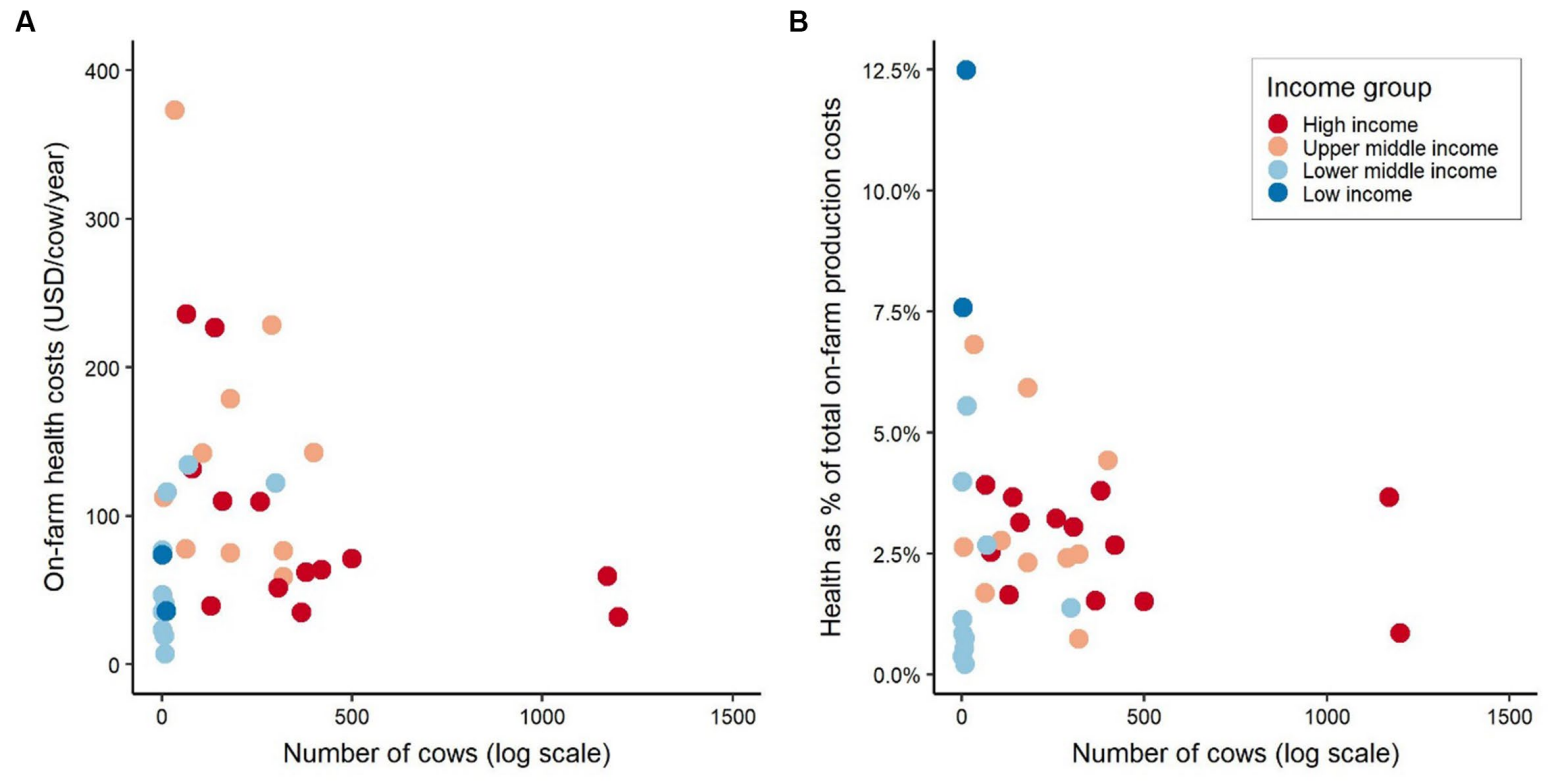
**(A)** On-farm health costs by number of cows and country income group, and **(B)** Health costs as proportion of total on-farm production costs by number of cows (log scale) and country income group. Costs values are presented in International Dollars (PPP adjusted values). Source: IFCN (59).

On-farm health costs per cow do not appear to change significantly, e.g., staying below 100 USD, based on the herd size, except for herds of between 100 and 500 animals for which health costs per cow seem to increase in some countries (Figure 9A). This relationship becomes more evident in Figure 9B, in which health costs as a proportion of total production costs are compared to the number of cows on farms. This figure also shows that low-income countries, i.e., Uganda, with small herds (13 or less cow) appear to spend a relatively high proportion of their total cost per cow on health compared to other countries.

### 3.7. Heath costs vs. losses of cows

**Figure 10:**
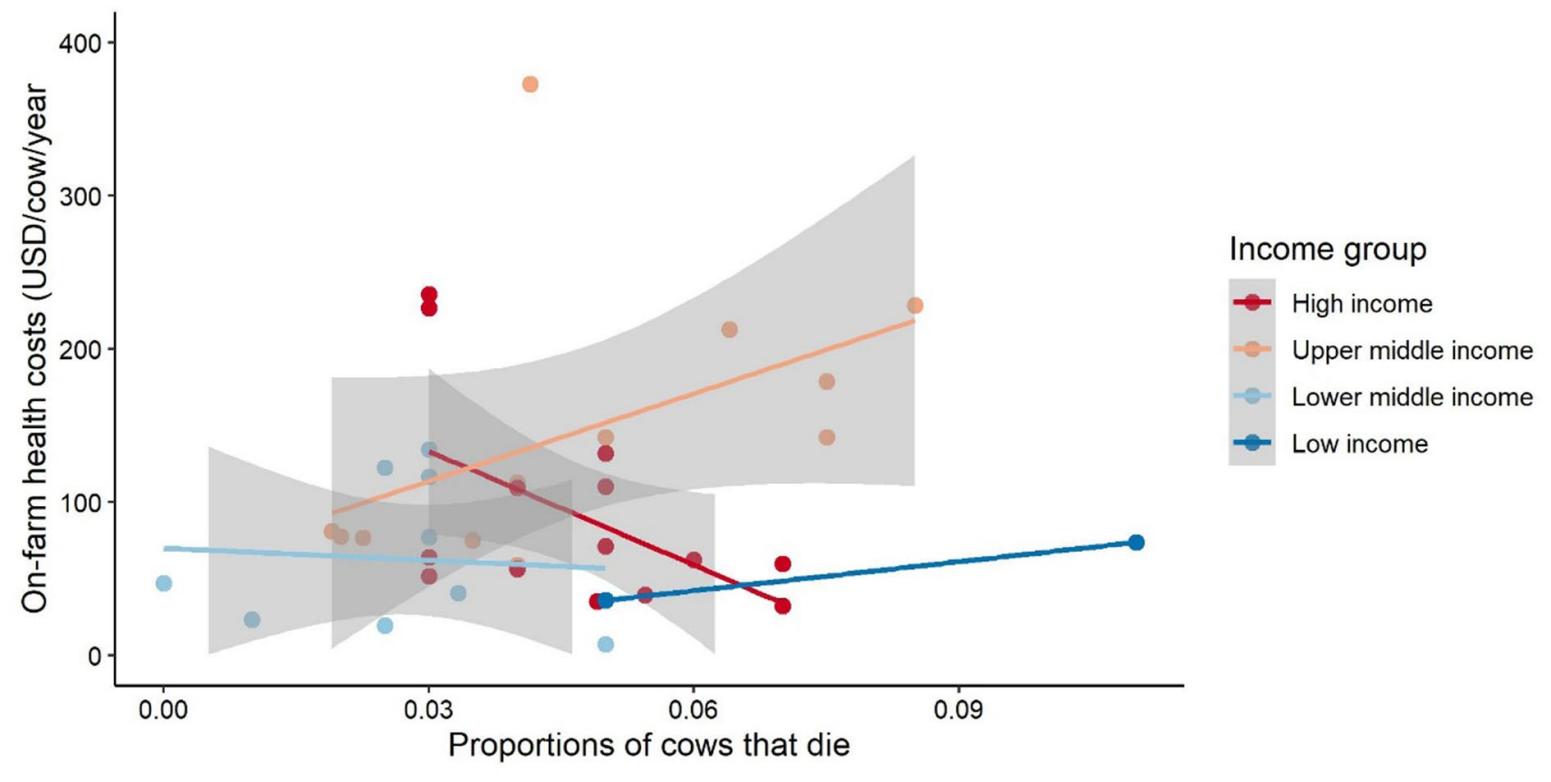
Health costs by proportion of cows that died. The gray area represents the confidence interval around the smoothed geometric mean. The smaller the grey area around the mean trend line the more similar the observations within a country income group and vice versa. Cost values are presented in International Dollars (PPP adjusted values). Source: IFCN (59).

A comparison of on-farm health costs with the proportion of cows that die (Figure 10) suggests that an increase in on-farm health costs may lead to a decrease in mortality (i.e., proportion of cows that die) for high-income countries. This trend is also observed for lower middle-income countries, but it is less clear. The opposite relationship was identified for upper middle-income countries and lower income countries, i.e., higher health expenses leading to higher mortality. An explanation for this result could be that the quality of, and access to, animal health care and monitoring only significantly increases once a country reaches a high-income level. Furthermore, external health support or directive such as national animal health programs (e.g., mandatory vs. reactive vaccination programs) may indirectly impact this relationship, which cannot be verified in the absence of data. Farm system specific aspects, e.g., health cost per cow and management of herds in fully confined systems may be different compared to pasture-based or semi-confined systems which may also indirectly be reflected in the results. Yet, caution should be used in generalizing these results since Figure 10 shows outliers to this trend. Additional results (e.g., on losses, assessment by fixed and variable cost types) are presented in the Supplementary material.

## 4. Discussion

The results presented in this study offer new insights into on-farm health expenditure patterns for dairy cattle across different countries and a comparison of animal health expenditures to other production cost types (e.g., feed, labor, capital), milk yields and cow losses. These are critical data for the GBADs program and ones that are not easily accessible.

A key finding suggests that on-farm health expenditures across all countries are relatively low, ranging between 0.2–12.5% of the total production costs per cow *per annum* (Figure 5), which is similar to previous reports [e.g., (60, 61)]. Farms in low-income countries (Uganda) tend to have lower overall expenditure and spend a higher proportion of their total costs per cow per year on animal health (e.g., 10%) than farms in higher income countries (Figure 5). These findings are supported by Waiswa and Günlü (51) who found that veterinarian, drugs, acaricides, and vaccination costs combined can take up an even higher share, i.e., up to 24.9%, of total production costs for Ugandan dairy farms.

The results also indicate that the means of production costs can take up to 90% of the total costs for intensive feedlot dairy systems (Figure 3), where most of the feed is purchased. Means of production costs are also likely to be high in systems where feed is produced on-farm with high levels of inputs such as seed, fertilizer, and pest/weed control. This aligns with findings by Ruviaro et al. (62), who showed that feed costs alone (which were here treated as a part of means of production costs) can take up to 87% of the total production costs in semi-confined feedlot systems in Brazil. However, the analysis revealed that means of production costs can also be as low as 9–29% in low-input systems, e.g., for the Uganda farm types A and B. Again, this finding aligns with Waiswa and Günlü (51) who report that feed costs for dairy farms in Uganda take a share of 11.4% of the total production costs. This is likely because dairy cattle in Uganda are predominantly managed in extensive grazing systems with much lower inputs compared to dairy farms in higher income countries (63, 64).

A further finding from the analysis was that the total production costs per cow appear to increase with the country’s wealth. This may not be surprising since with increasing wealth of a country, higher quality production inputs may become available, but these may also be more expensive than in less wealthy countries (e.g., opportunity cost of land, cost of higher quality feed, advanced equipment and machinery) [e.g., (65, 66)].

The results show interesting patterns with respect to countries’ income status. For example, total on-farm costs (Figure 4A) and on-farm health costs (Figures 5, 10) were higher for farms in upper middle-income countries than both lower middle-income countries and high-income countries. This finding may be attributed to the farm types, e.g., very high number of cows, intense feedlot systems, in the upper middle-income country cluster (i.e., Brazil, China) and overrepresentation of these by including four farm types for Brazil and China in the data set in comparison to Argentina and Colombia for which only two farm types were available. Hence, the findings for the upper middle-income country category may be due to a country selection bias (e.g., high milk volume producing countries) and should be interpreted cautiously. A larger sample could offer more robust and clearer results about these aspects.

While this study offers insights into the value of systematically collected on-farm cost data for a global assessment of investments into dairy animal health, there are data gaps that should be addressed in future. For example, the lack of data for low-income countries, where the burden of animal disease has likely higher social and economic implications, is a concern. Furthermore, data that disaggregates on-farm health expenditures into different components should be collected, together with health management practices (e.g., frequency of animal vaccinations, veterinary health consultations) and prevailing diseases (see Supplementary material). Such information would assist the modeling of disease spread (e.g., nationally, regionally, and globally) and their impact on dairy herds. It would also provide the opportunity to measure the socio-economic impact of cattle diseases and their management as a potential basis for national and international investments in improved disease prevention. These data needs align and complement the list of data needs proposed by other authors such as Perry et al. (17) and Waiswa et al. (50).

Animal health is a public good since it can affect global human food security and human health (67–69). Hence, collected data on animal health aspects should be accessible for researchers and policy makers at a national and global scale as a basis for decision making. Currently, data sets that describe dairy production systems, including animal health aspects (e.g., health expenses, disease prevalence) exist, but mostly in silos and are not collated and harmonized. These data sets are typically inaccessible or only accessible at a cost for public good research purposes. This is a barrier to gaining an improved understanding about global on-farm animal health investments. Therefore, effective collaborations about the collection, analysis, and use of on-farm animal health data (including the transfer of technologies, methods, skills in developing data collection processes) need to be developed between the key stakeholders (e.g., research institution, industry associations, NGOs, private companies, national governments, and intergovernmental organizations). While establishing these collaborations may appear to be challenging, the Covid-19 pandemic has demonstrated that such partnerships between key stakeholders (e.g., pharmaceutical companies, national governments, World Health Organization and research institutions) can be effective if public human health is at risk [e.g., (70, 71)]. Learnings from this experience should be adapted to the global animal health/One Health context to avoid risks due to animal diseases for global food security and human health (e.g., zoonosis) (67, 69, 71).

A limitation of this study is the small sample size of countries and the lack of time series data for each country which affects the robustness and generalization of results (e.g., missing trends). A larger data set would offer the opportunity to include more advanced analytical approaches to establish potential causes for the observed production cost structures. This would also allow an assessment of on-farm costs by agri-ecological zone and production classification system. Data about on-farm dairy herd age structure, e.g., number of animals by age and use, as well as age and use specific production costs of the animals in the dairy herd would have been beneficial for more detailed health investment analysis.

A further limitation is the use of production cost data for 2021, which was a year in which Covid-19 was prevailing. Implications of Covid-19 restrictions, e.g., social distancing, labor shortages, limited logistics options, may have affected production costs and the availability of input factors. This may imply that the structure of on-farm costs may be different for this production year compared to previous production years. This highlights the need to compare expenses for on-farm animal health investments over time and assess changes and drivers for changes (e.g., policies, subsidized medicine, human pandemics).

Moreover, national animal health programs that provide financial incentives for on-farm animal health management (e.g., subsided medicine and vaccines) likely vary across countries and may affect on-farm health expenditures differently. This has not been included in the analysis but offers scope for future research. For example, a comparison of the 15 national animal health management strategies and policies, including investments in prevention and management of diseases, could provide insights to how national health programs may influence on-farm health expenditures and farmers’ decision making. This aligns with the research needs identified by Capper and Williams (5), e.g., the need to better understand producers’ and veterinarians’ perceptions and behaviors toward the disease management. An extension to the present work could identify potential gaps in countries’ governance of animal diseases and options how to address these as a global community with an interest in animal health and food security.

## 5. Conclusion

This study provides a proof of concept, using a subset of a global farm comparison dataset to demonstrate the value of systematically collected data about on-farm health expenditure and comparisons across countries. Such information offers insights into farm production cost structures, which can be useful for national governments and intergovernmental organizations to identify investment gaps in animal health as a public good that needs to be addressed through targeted policies. For example, our analysis highlights an imbalance in on-farm expenditure, with farms in low-income countries investing proportionally more in animal health compared to farms in higher-income countries. We also highlight data gaps, both in the geographical spread and diversity of farms surveyed, and the types of data collected, e.g., on-farm dairy herd age structure, prevailing diseases their management, disaggregation of heath expenses at national or even sub-national scales, which limit our ability to adequately corelate on-farm investments in animal health with animal health and productivity outcomes.

## Data availability statement

The data analyzed in this study is subject to the following licenses/restrictions: Data sharing agreement with data owner prevents the sharing of data with third parties. Requests to access these datasets should be directed to Name of data owner: IFCN; Website: https://ifcndairy.org/; Email: info@ifcndairy.org.

## Ethics statement

The studies involving humans were approved by CSIRO ethics and privacy committee (approval number 054/22). The studies were conducted in accordance with the local legislation and institutional requirements. The participants provided their written informed consent to participate in this study.

## Author contributions

PS: Conceptualization, Data curation, Formal analysis, Investigation, Methodology, Project administration, Software, Validation, Visualization, Writing – original draft. CGF: Conceptualization, Data curation, Formal analysis, Investigation, Writing – original draft. DM: Funding acquisition, Resources, Supervision, Writing – review & editing. MH: Funding acquisition, Supervision, Writing – review & editing.

## Footnotes

^1^ https://animalhealthmetrics.org/

## References

[1] Lagrange V Whitsett D Burris C. Global market for dairy proteins. J Food Sci. (2015) 80:A16–22. doi: 10.1111/1750-3841.12801, PMID: 25757893

[2] Britt JH Cushman RA Dechow CD Dobson H Humblot P Hutjens MF. Perspective on high-performing dairy cows and herds. Animal. (2021) 15:100298. doi: 10.1016/j.animal.2021.100298, PMID: 34266782

[3] Gauly M Bollwein H Breves G Brügemann K Dänicke S Das G. Future consequences and challenges for dairy cow production systems arising from climate change in Central Europe - a review. Animal. (2013) 7:843–59. doi: 10.1017/S1751731112002352, PMID: 23253935

[4] Wells SJ Ott SL Hillberg Seitzinger A. Key health issues for dairy cattle—new and old. J Dairy Sci. (1998) 81:3029–35. doi: 10.3168/jds.S0022-0302(98)75867-9 9839242

[5] Capper JL Williams P. Investing in health to improve the sustainability of cattle production in the United Kingdom: a narrative review. Vet J. (2023) 296:105988. doi: 10.1016/j.tvjl.2023.105988, PMID: 37150316

[6] Bar D Tauer LW Bennett G González RN Hertl JA Schukken YH. The cost of generic clinical mastitis in dairy cows as estimated by using dynamic programming. J Dairy Sci. (2008) 91:2205–14. doi: 10.3168/jds.2007-0573, PMID: 18487643

[7] Deka RP Magnusson U Grace D Randolph TF Shome R Lindahl JF. Estimates of the economic cost caused by five major reproductive problems in dairy animals in Assam and Bihar, India. Animals. (2021) 11:3116. doi: 10.3390/ani11113116, PMID: 34827848 PMC8614483

[8] Dolecheck K Bewley J. Animal board invited review: dairy cow lameness expenditures, losses and total cost. Animal. (2018) 12:1462–74. doi: 10.1017/S1751731118000575, PMID: 29557318

[9] Liang D Arnold LM Stowe CJ Harmon RJ Bewley JM. Estimating us dairy clinical disease costs with a stochastic simulation model. J Dairy Sci. (2017) 100:1472–86. doi: 10.3168/jds.2016-11565, PMID: 28012631

[10] Puerto MA Shepley E Cue RI Warner D Dubuc J Vasseur E. The hidden cost of disease: I. Impact of the first incidence of mastitis on production and economic indicators of primiparous dairy cows. J Dairy Sci. (2021) 104:7932–43. doi: 10.3168/jds.2020-19584 33865582

[11] Ott SL Wells SJ Wagner BA. Herd-level economic losses associated with Johne's disease on us dairy operations. Prev Vet Med. (1999) 40:179–92. doi: 10.1016/S0167-5877(99)00037-9, PMID: 10423773

[12] Rasmussen P Barkema HW Mason S Beaulieu E Hall DC. Economic losses due to Johne's disease (paratuberculosis) in dairy cattle. J Dairy Sci. (2021) 104:3123–43. doi: 10.3168/jds.2020-19381 33455766

[13] Huijps K Lam TJGM Hogeveen H. Costs of mastitis: facts and perception. J Dairy Res. (2008) 75:113–20. doi: 10.1017/S0022029907002932, PMID: 18226298

[14] Alhaji NB Amin J Aliyu MB Mohammad B Babalobi OO Wungak Y. Economic impact assessment of foot-and-mouth disease burden and control in pastoral local dairy cattle production systems in northern Nigeria: a cross-sectional survey. Prev Vet Med. (2020) 177:104974. doi: 10.1016/j.prevetmed.2020.104974, PMID: 32240887

[15] Boisvert RN Kay D Turvey CG. Macroeconomic costs to large scale disruptions of food production: the case of foot- and-mouth disease in the United States. Econ Model. (2012) 29:1921–30. doi: 10.1016/j.econmod.2012.06.007

[16] Knight-Jones TJD Rushton J. The economic impacts of foot and mouth disease – what are they, how big are they and where do they occur? Prev Vet Med. (2013) 112:161–73. doi: 10.1016/j.prevetmed.2013.07.013, PMID: 23958457 PMC3989032

[17] Perry BD Aklilu Gebreyes Y Hailemariam S Legese G Smyth K Peters AR. A multi-stakeholder participatory pilot study of the data demands of the future Ethiopian dairy sector. Gates Open Res. (2022) 6:51. doi: 10.12688/gatesopenres.13594.2 35923864 PMC9296832

[18] Rushton J Huntington B Gilbert W Herrero M Torgerson PR Shaw APM. Roll-out of the global burden of animal diseases programme. Lancet. (2021) 397:1045–6. doi: 10.1016/S0140-6736(21)00189-6 33549170

[19] The World Bank. Country classification–World Bank counrty and lending groups. Washington, DC: The World Bank (2021).

[20] FAO. Faostat. Rome, Italy: Food and Agriculture Organization of the United Nations (2023).

[21] Hemme T. Dairy report 2014. Kiel, Germany: International Farm Comparison Network (IFCN) (2014).

[22] Hemme T Uddin MM Ndambi OA. Benchmarking cost of Milk production in 46 countries. J Rev Glob Econ. (2014) 3:254–70. doi: 10.6000/1929-7092.2014.03.20

[23] The World Bank. Purchasing power parities – Putting a global public good to work in socioeconomic analyses. Washington, DC: The World Bank Group (2022).

[24] The World Bank. Ppp conversion factor, Gdp (Lcu per international $). World development indicators database. Washington, DC: The World Bank Group (2022).

[25] Lazzarini B Baudracco J Tuñon G Gastaldi L Lyons N Quattrochi H. Review: Milk production from dairy cows in Argentina: current state and perspectives for the future. Appl Anim Sci. (2019) 35:426–32. doi: 10.15232/aas.2019-01842

[26] Sheng Y Chancellor W Jackson T. Deregulation reforms, resource reallocation and aggregate productivity growth in the Australian dairy industry. Aust J Agric Resource Econ. (2020) 64:477–504. doi: 10.1111/1467-8489.12351

[27] Dairy Australia. In focus 2021: The Australian dairy industry. Southbank, Australia: Dairy Australia Limited (2021).

[28] Datta AK Haider MZ Ghosh SK. Economic analysis of dairy farming in Bangladesh. Trop Anim Health Prod. (2019) 51:55–64. doi: 10.1007/s11250-018-1659-7 30003526

[29] Uddin MM Akter A Khaleduzzaman ABM Sultana MN Hemme T. Application of the farm simulation model approach on economic loss estimation due to coronavirus (Covid-19) in Bangladesh dairy farms–strategies, options, and way forward. Trop Anim Health Prod. (2020) 53:33. doi: 10.1007/s11250-020-02471-8, PMID: 33230604 PMC7682769

[30] Hossain S Jahan M Khatun F. Current status of dairy products in Bangladesh: a review on supply and utilization. Int J Bus Manage Soc Res. (2022) 11:609–18. doi: 10.18801/ijbmsr.110222.65

[31] Balcão LF Longo C Costa JHC Uller-Gómez C Filho LCPM Hötzel MJ. Characterisation of smallholding dairy farms in southern Brazil. J Anim Prod Sci. (2017) 57:735–45. doi: 10.1071/AN15133

[32] Daros RR Hötzel MJ Bran JA Leblanc SJ Von Keyserlingk MAG. Prevalence and risk factors for transition period diseases in grazing dairy cows in Brazil. Prev Vet Med. (2017) 145:16–22. doi: 10.1016/j.prevetmed.2017.06.004, PMID: 28903871

[33] Mc Geough EJ Little SM Janzen HH Mcallister TA Mcginn SM Beauchemin KA. Life-cycle assessment of greenhouse gas emissions from dairy production in eastern Canada: a case study. J Dairy Sci. (2012) 95:5164–75. doi: 10.3168/jds.2011-5229 22916922

[34] Van Kooten GC. Reforming Canada’s dairy sector: Usmca and the issue of compensation. Appl Perspect Policy. (2020) 42:542–58. doi: 10.1093/aepp/ppy038

[35] Charlebois S Bowdridge E Lemieux J-L Somogyi S Music J. Supply management 2.0: a policy assessment and a possible roadmap for the Canadian dairy sector. Foods. (2021) 10:964. doi: 10.3390/foods10050964, PMID: 33924953 PMC8145998

[36] Li XL Yuan QH Wan LQ He F. Perspectives on livestock production systems in China. Rangeland J. (2008) 30:211–20. doi: 10.1071/RJ08011

[37] Huang J Xu C-C Ridoutt BG Liu J-J Zhang H-L Chen F. Water availability footprint of milk and milk products from large-scale dairy production systems in Northeast China. J Clean Prod. (2014) 79:91–7. doi: 10.1016/j.jclepro.2014.05.043

[38] Carulla JE Ortega E. Dairy production Systems of Colombia: challenges and opportunities. Latin Am Arch Anim Prod. (2016) 24:83–7.

[39] Kumar A Parappurathu S Joshi P. Structural transformation in dairy sector of India. Struct Transform Dairy Sect India. (2013) 26:209–20.

[40] Landes M Cessna J Kuberka L Jones K. India’s dairy sector: Structure, performance, and prospects. United States: United States Department of Agriculture (USDA) (2017).

[41] Susanty A Bakhtiar A Puspitasari NB Susanto N Handjoyo DKS. The performance of dairy supply chain in Indonesia: a system dynamics approach. Int J Product Perform Manag. (2019) 68:1141–63. doi: 10.1108/IJPPM-09-2018-0325

[42] Umberger W. The IndoDairy smallholder household survey (Ishs) from ‘farm-to-fact’ series. Adelaide, Australia: Univerity of Adelaide, The Centre for Global Food and Resources (2020).

[43] Apdini T Al Zahra W Oosting SJ De Boer IJM De Vries M Engel B. Understanding variability in greenhouse gas emission estimates of smallholder dairy farms in Indonesia. Int J Life Cycle Assess. (2021) 26:1160–76. doi: 10.1007/s11367-021-01923-z

[44] Onono JO Wieland B Rushton J. Productivity in different cattle production systems in Kenya. Trop Anim Health Prod. (2013) 45:423–30. doi: 10.1007/s11250-012-0233-y 22820942

[45] Kibiego MB Lagat JK Bebe BO. Competitiveness of smallholder Milk production Systems in Uasin Gishu County of Kenya. J Econ Sustain Dev. (2015) 6:39–45.

[46] Odero-Waitituh JA. Smallholder dairy production in Kenya: a review. Livest Res Rural Dev. (2017) 29:29–47.

[47] Dairy Nz. New Zealand dairy statistics 2020–21. Hamilton, New Zealand: Livestock Improvement Corporation Limited and Dairynz Limited (2021).

[48] Foote KJ Joy MK Death RG. New Zealand dairy farming: milking our environment for all its worth. Environ Manag. (2015) 56:709–20. doi: 10.1007/s00267-015-0517-x, PMID: 25900603

[49] Kirunda H Kabi F Muwereza N Kabuuka T Magona JW Lukwago G. Knowledge and perceptions of smallholder dairy farmers of cattle disease burdens in selected agro-ecological zones of Uganda. Uganda J Agric Sci. (2012) 13:107–16.

[50] Waiswa D Günlü A Mat B. Development opportunities for livestock and dairy cattle production in Uganda: a review. Res J Agric Forest Sci. (2021) 9:18–24.

[51] Waiswa D Günlü A. Economic analysis of dairy production in Uganda, a case study on the performance of dairy cattle enterprises in southwestern Uganda. Asian J Agric. (2022) 6:61–7. doi: 10.13057/asianjagric/g060202

[52] Arnott G Ferris CP O’connell NE. Review: welfare of dairy cows in continuously housed and pasture-based production systems. Animal. (2017) 11:261–73. doi: 10.1017/S1751731116001336, PMID: 27364762

[53] Wilkinson JM Lee MRF Rivero MJ Chamberlain AT. Some challenges and opportunities for grazing dairy cows on temperate pastures. Grass Forage Sci. (2020) 75:1–17. doi: 10.1111/gfs.12458, PMID: 32109974 PMC7028026

[54] Khanal AR Gillespie J Macdonald J. Adoption of technology, management practices, and production systems in us milk production. J Dairy Sci. (2010) 93:6012–22. doi: 10.3168/jds.2010-3425, PMID: 21094776

[55] Von Keyserlingk MAG Martin NP Kebreab E Knowlton KF Grant RJ Stephenson M. Invited review: sustainability of the us dairy industry. J Dairy Sci. (2013) 96:5405–25. doi: 10.3168/jds.2012-6354 23831089

[56] Fariña SR Chilibroste P. Opportunities and challenges for the growth of milk production from pasture: the case of farm systems in Uruguay. Agric Syst. (2019) 176:102631. doi: 10.1016/j.agsy.2019.05.001

[57] Méndez MN Chilibroste P Aguerre M. Pasture dry matter intake per cow in intensive dairy production systems: effects of grazing and feeding management. Animal. (2020) 14:846–53. doi: 10.1017/S1751731119002349, PMID: 31650937

[58] Stirling S Fariña S Pacheco D Vibart R. Whole-farm modelling of grazing dairy systems in Uruguay. Agric Syst. (2021) 193:103227. doi: 10.1016/j.agsy.2021.103227

[59] IFCN. 2021 dairy production system data for selected countries (obtained under a confidential data sharing agreement). Kiel, Germany: International Farm Comparison Network (IFCN) (2021).

[60] Heinrichs AJ Jones CM Gray SM Heinrichs PA Cornelisse SA Goodling RC. Identifying efficient dairy heifer producers using production costs and data envelopment analysis<sup>1</sup>. J Dairy Sci. (2013) 96:7355–62. doi: 10.3168/jds.2012-6488, PMID: 24054291

[61] Shamsuddin M Goodger WJ Hossein MS Azizunnesa Bennett T Nordlund K. A survey to identify economic opportunities for smallholder dairy farms in Bangladesh. Trop Anim Health Prod. (2006) 38:131–40. doi: 10.1007/s11250-006-4274-y, PMID: 17682598

[62] Ruviaro CF De Leis CM Florindo TJ De Medeiros Florindo GIB Da Costa JS Tang WZ. Life cycle cost analysis of dairy production systems in southern Brazil. Sci Total Environ. (2020) 741:140273. doi: 10.1016/j.scitotenv.2020.140273 32887019

[63] Balikowa D. *Dairy development in Uganda: A review of Uganda’s dairy industry*. Rome, Italy: TechnoServe/East Africa Dairy Development Project (Eadd). Gou/Fao Dairy Project, Tcp/Uga/3202(D). Food and Agriculture Organization of the United Nations (FAO). (2011).

[64] Mubiru SL Tenywa JS Halberg N Romney D Nanyeenya W Baltenweck I. Categorisation of dairy production systems: a strategy for targeting meaningful development of the systems in Uganda. Livest Res Rural Dev. (2007) 19:103–11.

[65] Balehegn M Duncan A Tolera A Ayantunde AA Issa S Karimou M. Improving adoption of technologies and interventions for increasing supply of quality livestock feed in low- and middle-income countries. Glob Food Sec. (2020) 26:100372. doi: 10.1016/j.gfs.2020.100372, PMID: 33324534 PMC7726233

[66] Holden ST. Fifty years of research on land tenure policies and land markets: what are the major lessons? In: Estudillo JP Kijima Y Sonobe T, editors. Agricultural development in Asia and Africa: Essays in honor of Keijiro Otsuka. Singapore: Springer Nature Singapore (2023)

[67] Brown C Havas K Bowen R Mariner J Fentie KT Kebede E. Animal health in a development context. Glob Food Sec. (2020) 25:100369. doi: 10.1016/j.gfs.2020.100369

[68] Garcia SN Osburn BI Jay-Russell MT. One health for food safety, food security, and sustainable food production. Front Sustain Food Syst. (2020) 4:1–20. doi: 10.3389/fsufs.2020.00001

[69] Mardones FO Rich KM Boden LA Moreno-Switt AI Caipo ML Zimin-Veselkoff N. The Covid-19 pandemic and global food security. Front Vet Sci. (2020) 7:578508. doi: 10.3389/fvets.2020.578508, PMID: 33240957 PMC7683609

[70] Nasem. Public private partnership responses to Covid-19 and future pandemics: Proceedings of a workshop in brief. Washington: National Academies of Sciences, Engineering, and Medicine (Nasem) (2020).

[71] Tille F Panteli D Fahy N Waitzberg R Davidovitch N Degelsegger-Márque A. Governing the public-private-partnerships of the future: learnings from the experiences in pandemic times. Eur Secur. (2021) 27:49–53.

