# Supplementary material for: On-farm investments into dairy cow health: evidence from 15 case study countries
Source: Front Vet Sci. 2023 Oct 31;10:1288199. doi: 10.3389/fvets.2023.1288199 (PMC10643693; doi:10.3389/fvets.2023.1288199)
Supplement: Supplementary file 1 [file Data_Sheet_1.pdf]

Supplementary Material

On-farm investments into dairy cow health: Evidence from 15 case study countries

Peggy Schrobback*, Carlos Gonzalez Fischer, Dianne Mayberry, Mario Herrero

***Correspondence:** Peggy Schrobback: peggy.schrobback@csiro.au

1 Purchasing power parity (PPP) conversion factors

Table S.1. Average market exchange rates for LCU to USD and PPP conversion rates for 2021

Country	Market exchange rate from LCU to USD*	PPP conversion rates#
Argentina	0.01060	43.13541
Australia	0.74897	1.57118
Bangladesh	0.01178	33.19160
Brazil	0.18684	2.53068
Canada	0.80287	1.32439
China	0.15471	4.07480
Colombia	0.00027	1567.44666
India	0.01349	21.28467
Indonesia	0.00007	5067.26820
Kenya	0.00891	46.40900
New Zealand	0.70877	1.65802
Uganda	0.00028	1309.50549
United Kingdom	1.37478	0.78401
United States of America	1.00000	1.00000
Uruguay	0.02308	30.86636

Notes: 'LCU' for local currency unit. *Average market exchange rate was derived as the average of the highest and lowest rate of a LCU against the USD in 2021. # The PPP for private consumption was used as a conversion rate except for Argentina and Uganda for which these rates were unavailable 2021. For these two countries GDP PPP conversion rate were used. Source: https://www.xe.com/currencyconverter/ for exchange rate conversion LCU to USD. The World Bank (2022) for PPP adjustments of LCUs into International $.

2 Additional results on losses

In addition to losses of cows, the IFCN data set provided information on losses of calves that are born dead, and losses of calves between birth and weaning (male and female). These were reported as proportions without information about the denominator, e.g., absolute number of calves born, and causes for losses.

The results suggest that the highest proportion of losses of cows within the data set was observed in extensive systems, e.g., Uganda-A, intensive systems, e.g., China-C, USA-C, and semi-intensive system, e.g., Argentina-A, Argentina-B, New Zealand-B (see Figure S.1). The lowest proportion of losses in the data set were recorded for India-A and India-B, China-B, Brazil-B and Brazil-D.

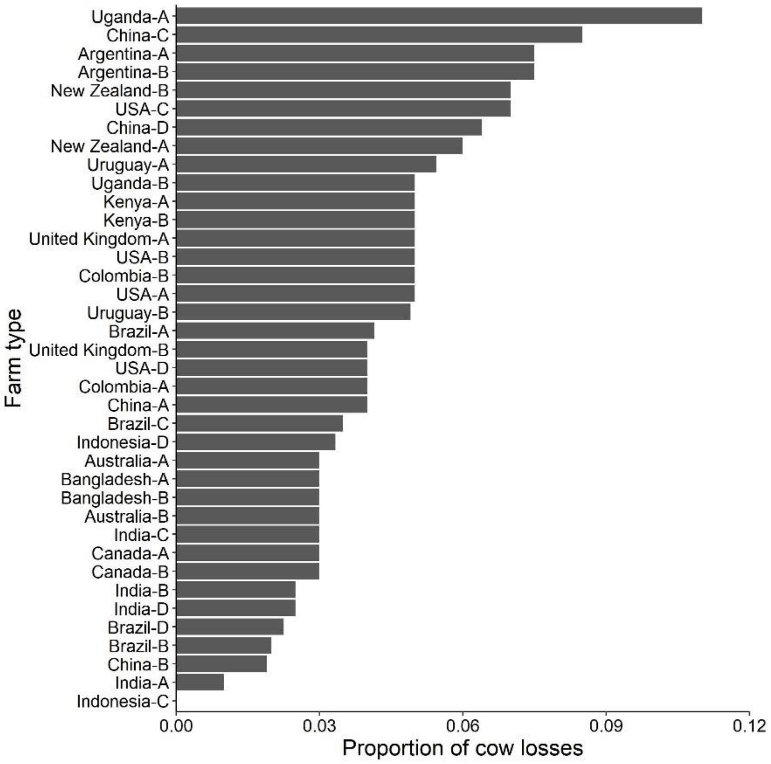

Figure S.1. Losses of cows by country and farm type. Notes: Information on cow losses for farm type A in Indonesia was missing in the data set. Losses are here considered as the proportion of cows that died during 2021. Source: IFCN (2021).

Figure S.2 shows the difference across country income groups for losses of cows that died, calves born dead, and calves that died before weaning (male and female). Low-income countries appear to have a higher loss proportion compared to all other country income groups. Lower middle-income, upper middle-income and high-income countries appear to have a similar median for the proportion of cows that die per annum, e.g., 3-5% (Figure S.2A). The results suggest that high income countries have the lowest rate of calves born dead, i.e., 5%, across all country groups, with lower middle- and upper middle-income countries following with about 6% and 8%, respectively (Figure S.2B). It should be noted that the proportion of calves born dead in low-income countries is significantly higher, e.g., 19% compared to all other countries. The results also imply that there is a relationship between higher country income and a lower proportion of calves dying before weaning (Figure S.2C). This result could be attributed to better animal health care being provided with rising country income.

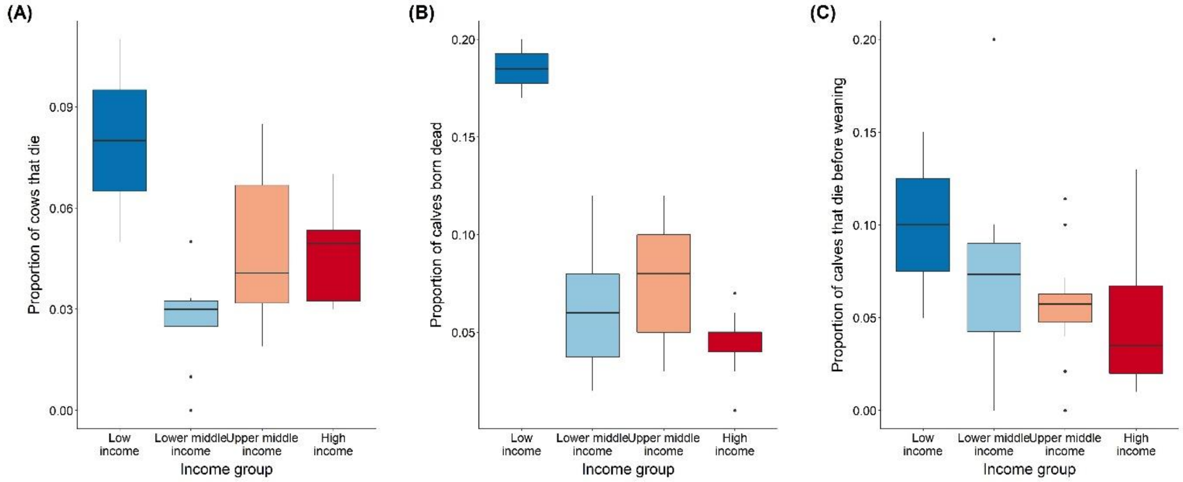

Figure S.2. (A) Proportions of cows that die by country income level, (B) Proportions of calves born dead by country income level, and (C) Proportion of calves that die before weaning (male and female) by country income level. Notes: Number of calves born was unavailable. Low-income group includes n=1, lower middle-income group includes n=4, upper middle-income group includes n=4, high-income group includes n=6. Source: IFCN (2021).

In Figure S.3A the annual proportion of cows that die are compared with the milk yield and Figure S.3B shows the relationship between the proportion of cows that die and the number to total cows (log scale) on farms. The results suggest that while the milk yield increases with higher income level, this is not reflected in the number of cows that died. Put differently, high milk yield producing countries, i.e., high-income countries, seem to exhibit a similar proportion of cow mortality compared to lower milk yield producing countries, except for low-income countries (Figure S.3B). The results suggest that the total number of cows on farms are not a predictor of the proportion of cows that died (Figure S.3B).

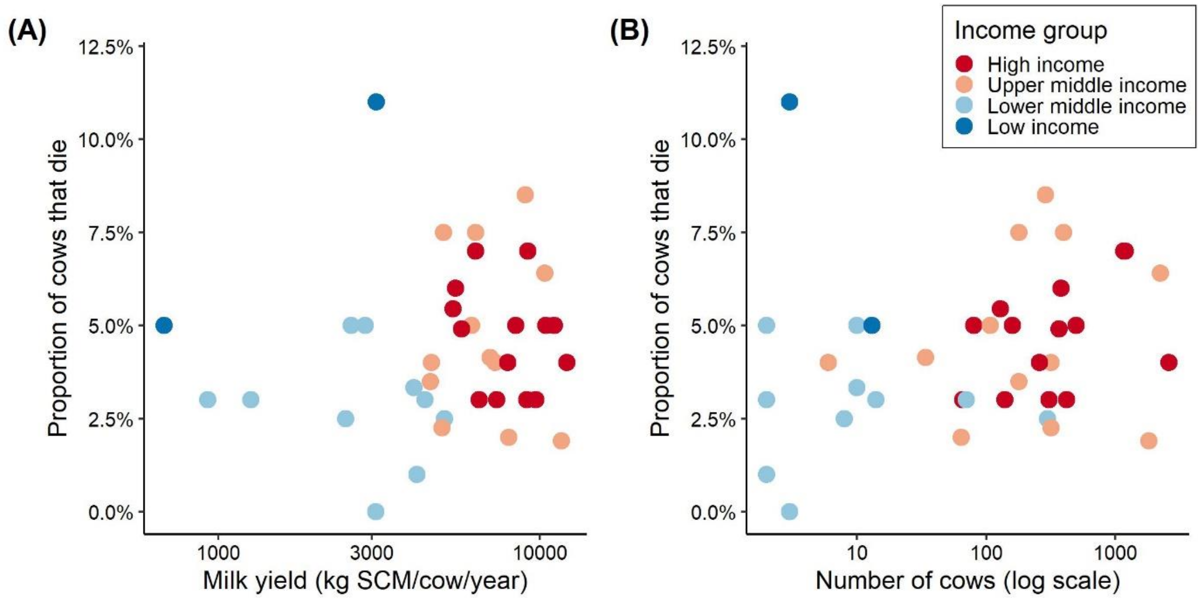

Figure S.3. (A) Cow losses compared to milk yield by country income group, and (B) Cow losses compared to the number of cows (log scale) on farm by country income group. Source: IFCN (2021).

3 Results for total production cost decomposition into fixed costs and variable costs

The total production costs can be decomposed into fixed costs and variable costs (see Figure S.4). Fixed costs are also known as indirect costs or overhead costs. These are farm expenses that are not dependent on the output units (e.g., quantity of animals produced) produced by the farm. Fixed costs are recurring costs to the farm which cannot be changed in the short run (note: the value of fixed costs is not permanently fixed; it can change over time). These costs must be paid regardless of output units being produced or not. Variable costs are costs that change as the output units that a farm produces changes. Variable costs of inputs can be varied in the short run.

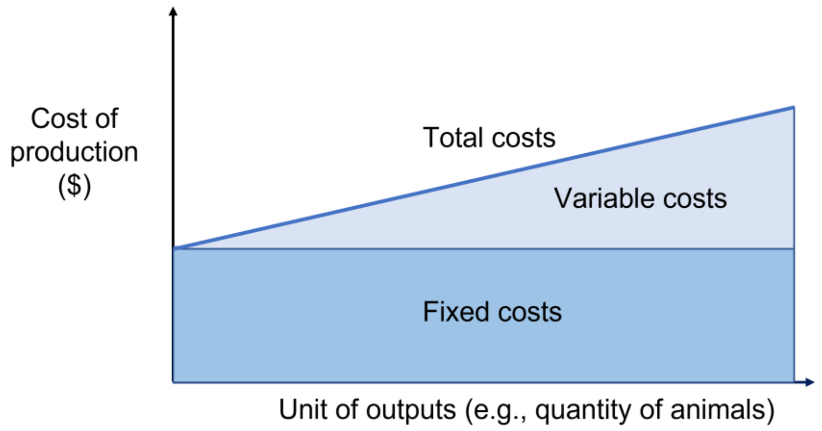

Figure S.4. Decomposition of total production costs.

Using the definition of fixed and variable costs as a baseline for on-farm production cost analysis, issues with the IFCN data set were encountered. For example, for some cost aggregates it is not possible to clearly identify if they are fixed or variable. This included 'Other inputs', 'Insurance taxes' which were aggregated to an extent, without details on what this included, that does not allow allocation into fixed and variable costs. However, as best as possible we categorized all available costs aggregates for all countries into fixed and variable as show in Table S.2.

Table S.2: Fixed and variable costs allocation for analysis

**Fixed costs**	**Variable costs**
Administrative costs (communication, marketing, accounting)	Feed (pasture, concentrate, supplements)
Family labor & family living	Health expenses (veterinary costs, drugs)
Buildings/facilities (capital, interest, depreciation)	Artificial insemination
Equipment/machinery (capital, interest, depreciation)	Electricity, power, water
Land (capita, taxes)	Fence & facilities repair and maintenance
Livestock (animal purchases)	Employed labor/contractor (incl. insurance, taxes, social security)
Farm insurance and taxes	Rent (e.g., pasture, machinery, hauling)
Capital	Fertilizer, seed, pesticides
	Fuel
	Other (milk supplies, herd testing)

Notes: *These are variable cost that are considered in the livestock production context. For other enterprises, this would be a fixed cost. Sources: Based on Özsayin (2019), Lacy (2009), Dhuyvetter (2001).

The results in Figure S.5 show the distribution of fixed and variable costs by farm type (Figure S.5A) and by fixed costs only (Figure S.5B). Figure S.5A suggests that variable costs dominate total production costs for all farm types except for farm type B in Uganda, which is an extensive grazing system. Results in Figure S.5B confirm that fixed costs proportion of total production costs for farm type B in Uganda were highest in the data set, i.e., lowest feed cost in due to communal grazing. The second lowest ranked farm type in the analysis was Canada-B. The lowest proportion of fixed costs in the data set were identified for the following farm types: China-C, Brazil-C, and Bangladesh-B.

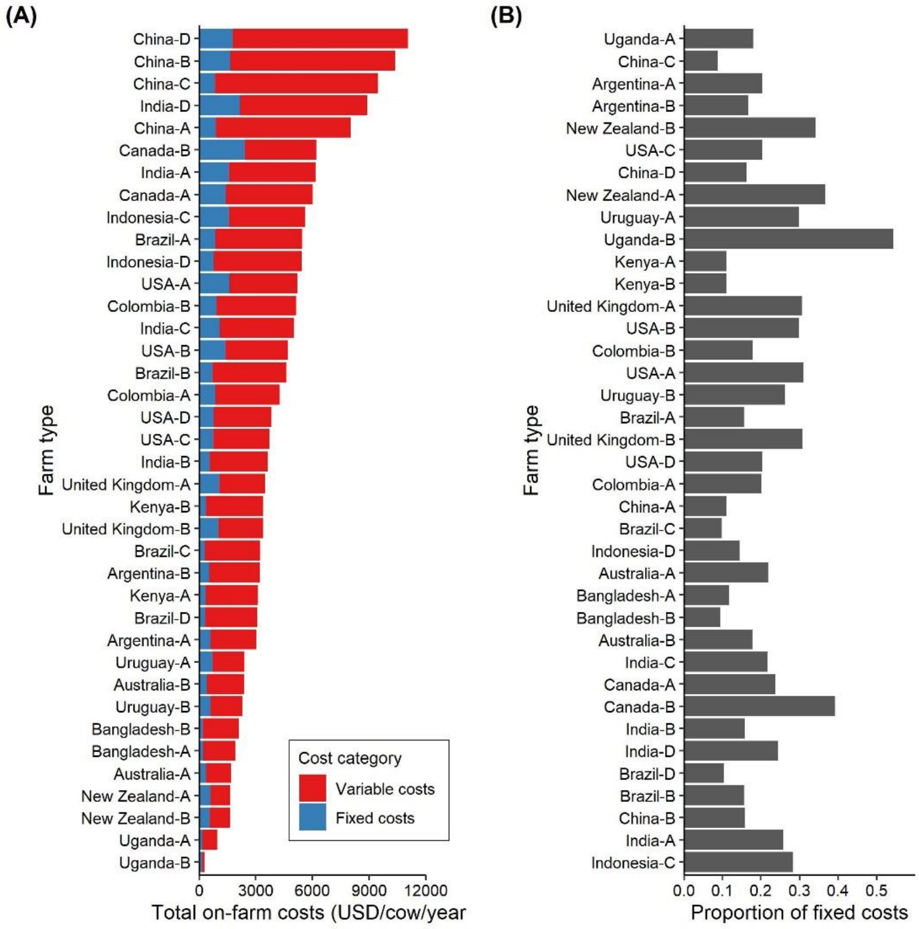

Figure S.5. (A) Distribution of fixed and variable costs by country and farm type, and (B) Distribution of proportion of fixed costs by country and farm type. Notes: Values are presented in International Dollars (i.e., USD value adjusted by PPP for each country). Source: IFCN (2021).

In Figure S.6 the findings for the decomposition of production costs into fixed and variable costs is shown by county income type. Similar as in Figure 4 in the main document, the results in Figure S.6 suggest that upper middle-income countries have the proportion highest variable costs (e.g., feed, intensive systems) across all country income categories. The proportion of variable costs compared to total costs in lower middle-income countries and high-income countries appears to be similar. Dairy farms in high-income countries appear to have the highest fixed costs.

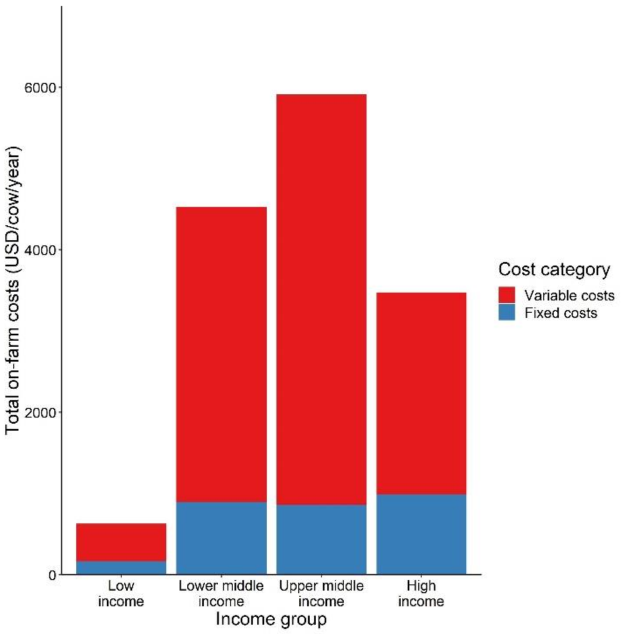

Figure S.6. Decomposition of production costs into average fixed and variable costs by country income type. Notes: Costs values are presented in International Dollars (PPP adjusted values). Source: IFCN (2021).

Figure S.7. provides further insights into the variability of a) on-farm fixed cost, b) on-farm variable costs, and c) fixed costs as a proposition of total on-farm costs by country income group.

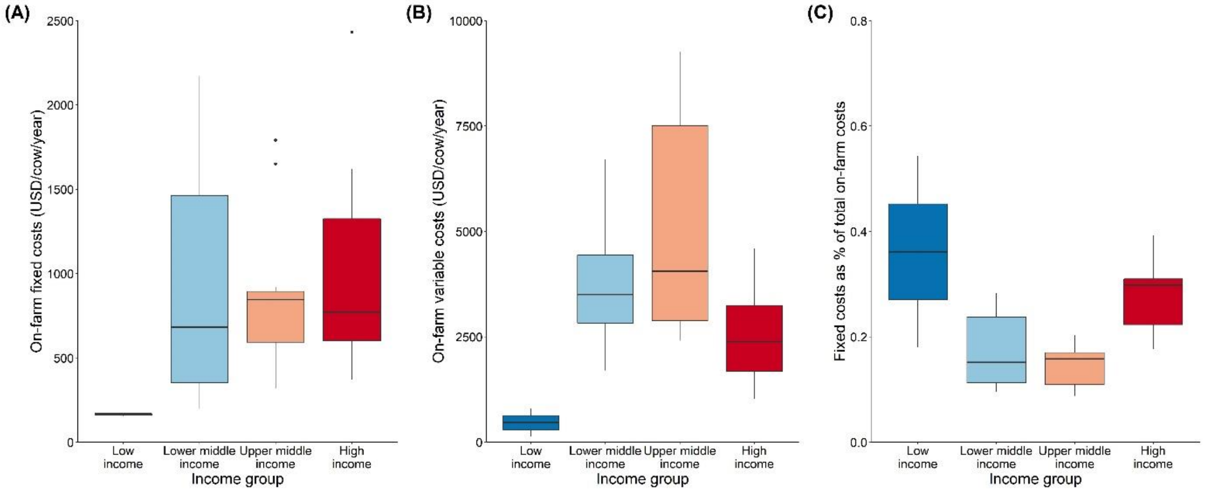

Figure S.7. (A) Variability of on-farm fixed cost by country income group, (B) Variability of on-farm variable costs by country income group, and (C) Variability of fixed costs as a proposition of total on-farm costs by country income group. Notes: Costs values are presented in International Dollars (PPP adjusted values). Source: IFCN (2021).

4 Expert interview questionnaire

Target group for expert interviews were dairy veterinarians, dairy extension officers, dairy scientists.

Background information about the purpose of the study was provided to participants prior to the interview.

Questions

First, I got a few questions about your role in the dairy sector:

- What is your current role within *[country name]* dairy sector? [Prompts: Dairy veterinarians, dairy extension officers, dairy scientists, other then please specify]
- How many herd visits (e.g., advice on productivity, treatment/prevention) per month have you conducted on average in 2021?
- How many years of experience do you have in this role or the dairy sector?

Now, let's talk about the dairy production system and production costs in your country:

- Could you please describe the most common or typical dairy farm type in your country in 2021? The is the farm type that represents the largest number of dairy farms in your country this could be based on:
    - Number of animals, type of calving patterns (e.g., seasonally, mixed systems), or feeding systems (e.g., pasture, grain/pasture, total mixed ration).
- What would you say was the average number of animals, including cows, dry cows, heifers, calves, bulls or male animals up to weaning, a typical dairy farm dairy production system in your country in 2021?
    - Of these animals, how many cows were there on average on a typical dairy farm?
- Could you please describe the most productive dairy farm type in term of milk yield in your country in 2021?
    - What would you say was the average number of animals, including cows, dry cows, heifers, calves, bulls or male animals up to weaning, on these highly productive dairy farms in 2021?
    - Of these animals, how many cows were there on average on these highly productive dairy farms?

Ok, we identified the typical and most productive dairy farm types in your country, the following questions will be about on-farm animal health costs and health management:

- Of the total production costs (e.g., feed, labor, infrastructure, reproduction, etc.) for a herd (e.g., cows, dry cows, calves, heifers) on your country's typical dairy farms, what would you say was the approximate proportion that was allocated to dairy animal health cost (e.g., drugs, vaccine, vet services) in 2021?
    - Would the on farm-health costs per animal be different for highly productive dairy farms compared to typical farms in your county? Is so, why?
    - What would you say were the total absolute annual average farm-level health costs per animal (e.g., cows, dry cows, calves, heifers) for typical dairy farms in your country?
- Of the total annual average health costs per dairy herd on a typical farm, how much would you say was spent on the following cost items as a proportion of total health costs:
    - Drugs and pharmaceuticals for prevention (i.e., vaccine, preventative measures) and treatment (i.e., reactive measures) of diseases: ________%

    - Veterinary consultancy visits (e.g., vet advice on herd health such as reproductive management, parasite control, heifer replacement, lameness management)): ________%
    - Reproduction (e.g., AI, pregnancy monitoring & assessment): ________% [If reproduction cost is treated as a separate cost compared to health costs, please advise the interviewer]
    - Any other costs that do not fall into these categories above (e.g., surgeries in case of calving difficulties)? Yes/No
        i. If yes, please specify these other costs and provide a proportion that accounts for these costs compared to the total health cost cow/dairy herd. ________%
- Do you expect these proportions to be different for the highly productive farm type? Yes/No
    - If yes, please provide respective proportions and comment on why they may be different?
- Can you break the estimate for 'drugs and pharmaceuticals' costs further down into prevention for disease prevention (i.e., preventative measures such as vaccines, testing disease prior to herd entry) and treatment (i.e., reactive measures)? Yes/No
    i. If yes, please provide proportions: _______% for prevention & _____% for treatment account for total drug and pharmaceutical costs.
    ii. If no, please explain why: ________________
- Do you expect these proportions to be different for the highly productive farm type? If so, why?
- Of the total costs for drugs and pharmaceuticals that are spend on a typical dairy farm, what is the proportion of costs allocated for anti-biotics and what is the proportion of costs allocated to other types of medicines? Do you expect these proportions to be different for the highly productive farm type? If so, why?
- Turning to anti-biotics (i.e., disease treatment, prevention of growth/productivity enhancement). How would you allocate on-farm costs for anti-biotics to these three areas of use:
    i. ___% disease prevention
    ii. ___% disease treatment
    iii. ___% animal growth enhancement.
- How is animal disease prevention and treatment handled in your country? For example, is it scheduled/planned according to seasons or is it reactive management to diseases occurrence?
- Are there any national programs in your country that offer dairy herd health costs for free or provide subsidies for animal disease prevention or treatment measures? Yes/No
    i. If yes, please specify what these measures are (e.g., medicine, vitamins, vet fees, AI, reproduction incentives): ________________
    ii. If yes, what are the implications for on-farm animal health cost? ________
- What is the most common reproductive method for cows in your country (e.g., AI, seasonal reproduction):___________? Would that be different for typical farms and highly productive farm type?

The last part of the interview focuses on cow diseases.

- Could you please list the three most common cow diseases in your country in 2021?
    1. ______________
    2. ______________
    3. ______________

- Could you please rank these diseases by prevalence?
    1.______________
    2.______________
    3.______________

- Could you please rank these diseases by total (prevention & treatment) expenditure in 2021? For example, what was the disease that is most costly to manage in your country?
    1.______________
    2.______________
    3.______________

This concludes the interview. Thank you.

5 Key learnings from conducting the expert interviews on the topic 'on-farm health expenses in the global dairy sector'

The questionnaire was initially tested with one dairy expert (veterinarians) in the USA and Australia, respectively. Their comments were used to refine he interview questionnaire (see above).

However, the research team encountered several issues in conducting 3-5 expert interviews in the 15 countries. These issues included:

a) Identification of participants who were sufficiently knowledgeable about on-farm finances (e.g., Bangladesh, Indonesia) and willing to share their insights (e.g., China, Australia) despite assistance from key national and international dairy association (e.g., WOHA, IDA, IFCN) in identifying experts,
b) A wide range of variance in the responses from the interviewed experts in most countries were identified which led to unreliable results; usually this would be addressed by conducting more interviews until the variance decreases but this was not possible due to the issue listed under a),
c) The terms 'disease prevention' and 'disease treatment' appeared to be understood differently in different countries and comparing responses would not have been meaningful across the 15 countries, and
d) Undertaking the interviews was a very time-consuming process due to different time zones, bad internet connections on the interviewees side (sometimes 2-3 attempts on different days were required to connect at sufficient bandwidth), participants accepted the meeting invitation but did not turning up at all or turned up 15-20 minutes late.

In summary, expert interviews as a data collection method may work well in different settings, e.g., data collection in only one country, prior knowledge about who the experts are and their willingness to share their knowledge, relative certainty that they are actual experts, but in the global context on this specific topic this data collection method appears to be less appropriate.

References

Dhuyvetter, K. C. (2001). *Fixed and Variable Cost Principles for Financial Planning (Fram Management Guide MF-1006)*. Kansas State University, Department of Agricultural Economics. Retrieved from https://farmanswers.org/Library/OpenItem/1837

IFCN. (2021). *2021 dairy production system data for selected countries (obtained under a confidential data sharing agreement)*. Kiel, Germany: International Farm Comparison Network (IFCN)

Lacy, R. C. (2009). *Understanding and Controlling Basic Cost Inputs into the Cow Herd (Florida Beef Cattle Short Course)*. Tifton: The University of Georgia. Retrieved from https://animal.ifas.ufl.edu/beef_extension/bcsc/2009/pdf/lacy_cow_herd.pdf

Özsayin, D. (2019). Economic analysis of fdairy cattle farms in Turkey: A case of Karacabey District. *Turkish Journal of Agriculture and Natural Sciences, 6*(4), 759-765. doi:https://doi.org/10.30910/turkjans.633588

The World Bank. (2022). *PPP conversion factor, GDP (LCU per international $)*. World Development Indicators Database. The World Bank Group. Retrieved from https://data.worldbank.org/indicator/PA.NUS.PPP
